# Optical and Magnetic Properties of Yellow Chromophore S_2_^•–^ in Ultramarine Pigments: CASPT2 and DFT Studies

**DOI:** 10.1021/acs.jpca.6c03753

**Published:** 2026-08-14

**Authors:** Paweł Rejmak, Mariusz Radoń, Piotr Pietrzyk

**Affiliations:** † 86906 Institute of Physics Polish Academy of Sciences, Al. Lotników 32/46, PL-02668 Warsaw, Poland; ‡ 37799 Jagiellonian University, Faculty of Chemistry, Gronostajowa 2, PL-30-387 Kraków, Poland

## Abstract

Disulfur radical anion S_2_^•–^ attracts attention as a yellow chromophore, orange emitter, and participant in many industrially relevant chemical and electrochemical processes, e.g., in metal–sulfur batteries. The reactive S_2_^•–^ radical can be stabilized by embedding in protective matrices, such as ultramarine pigments, where it accompanies other sulfur-radical species, mostly S_3_^•–^. Despite the apparent simplicity of S_2_^•–^, the assignment of its electron paramagnetic resonance (EPR) signal in ultramarines remains controversial. To elucidate this topic, we computationally investigate S_2_^•–^ embedded in cluster models of aluminosilicate cages in ultramarines. We find that the optical properties of S_2_^•–^ can be reliably treated using time-dependent density functional theory (DFT). However, to correctly predict the *g* tensor of either isolated or embedded S_2_^•–^, we find it necessary to employ the multiconfigurational complete active space method (CASSCF/CASPT2), combined with a variational treatment of strong spin–orbit coupling (SOC), stemming from orbital near-degeneracy. The *g* tensor of S_2_^•–^ in ultramarine is predicted to be strongly anisotropic, nearly axial, and notably environment-dependent, which may contribute to the difficulties in its unambiguous experimental characterization. Our results help clarify the assignment of the S_2_^•–^ EPR signal, elucidating some partially correct experimental data and identifying other interpretations as erroneous. This study should also be relevant for modeling other dichalcogenide radicals embedded in solid-state matrices.

## Graphical abstract

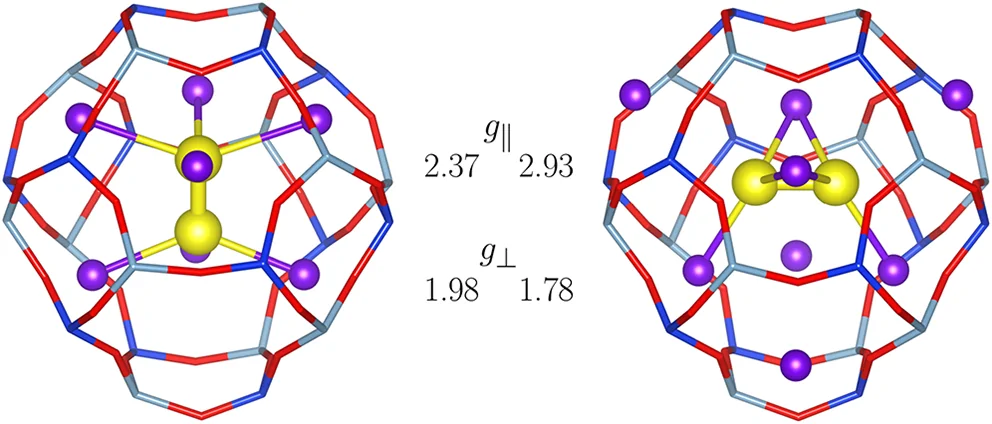

## 1 Introduction

Anionic polysulfur radicals play a major role in many sulfur→sulfide redox processes,^1,2^ taking place in metal–sulfur batteries,^3–8^ catalytic^9^ and photocatalytic^10,11^ organic synthesis, quantum dot-sensitized solar cells,^12^ Earth’s crust,^13^ and living cells.^14^ These radicals, although reactive and hence short-living under ambient conditions, can be stabilized by entrapment in protective matrices, the best known examples of such systems being ultramarine (UM) pigments. The most common UMs, occurring in nature as mineral lazurite, are blue pigments, consisting of S_3_^•–^ chromophore encapsulated in aluminosilicate nanocages of sodalite type lattice. Many synthetic analogues of UMs, based on different aluminosilicate hosts and polychalcogen chromophores, are also known.^15,16^

Disulfur radical anion S_2_^•–^ has been observed within defected alkali halides,^17–22^ neon matrix,^23^ hydrothermal fluids,^24^ borate glasses,^25,26^ and UMs,^15,16,27^ where it can be identified by the absorption of visible and infrared radiation at about 400 nm (yellow chromophore) and 590 cm^–1^, respectively. There is an increasing attention paid to S_2_^•–^ bearing aluminosilicates, due to their strong orange luminescence (at about 650 nm) from the aforementioned disulfur radical,^28–32^ and photochromic properties of these materials,^30,33–36^ which are attributed, based on computational studies,^36–40^ to the S_2_^2–^↔S_2_^•–^ + e^–^ process.

Although the optical properties of S_2_^•–^ ions are well understood, the analysis of their electron paramagnetic resonance (EPR) spectra is a more debated issue. The EPR measurements for S_2_^•–^ radical embedded in defected alkali halides showed large anisotropy of *g* tensor principal values and their strong dependence on the local environment, with the isotropic *g* value (*g*_iso_) spanning the range 1.75–2.15.^18,19,21,22^ The EPR experimental data for these systems were successfully reproduced at the density functional theory (DFT) level using large cluster models.^41,42^ However, the detection and assignment of S_2_^•–^ EPR signal in UMs and related materials, where several types of paramagnetic species are present, is more complicated. Initially, the broad signal with *g*_iso_ about 2.4 was identified as stemming from S_2_^•–^ ion,^43^ but it was later ascribed to Fe­(III) impurities.^44^ Another tentative assignment of a broad EPR band with *g*_iso_ of 2.01 to S_2_^•–^ ion is questionable, due to the isotropy of this signal.^16^ Only in 2011 Raulin et al. in a carefully conducted multitechnique study on UMs showed that a broad signal, resolvable at low temperature into components of *g*_1/2/3_ = 2.69/2.03/1.86, should be attributed to S_2_^•–^ radical.^45^ One of the authors of the present work, in his computational paper focusing on S_3_^•–^ radicals in UMs, performed preliminary DFT cluster studies on S_2_^•–^ ions.^46^ He found that in contrast to S_3_^•–^ ion, whose properties are very alike in the gas phase and different models of UMs, the corresponding DFT data (*g*_iso_ and the energy of optical transitions) for S_2_^•–^ radical are strongly dependent on the structural model and exchange-correlation functional. These results pointed out that the S_2_^•–^ signal in ultarmarines might be sensitive to the local environment but also revealed possible DFT problems with the proper description of splitting the partially unoccupied π*­(3*p*_*x*_, 3*p*_*y*_)^3^ level, determining the magnetic and optical properties of the discussed radical. Consequently, the need for further computational studies was postulated, involving advanced electronic structure methods beyond DFT.

This work aims to elucidate the EPR and UV–Vis signals of S_2_^•–^ radicals embedded in aluminosilicate matrices by employing both DFT and multiconfigurational complete active space (CASSCF/CASPT2) calculations. The calculations will be performed for realistic cluster models, and the effect of the model (type and size) will be investigated. Different treatments of the spin–orbit coupling in quasi-degenerate electronic states of S_2_^•–^ radicals will be discussed. The obtained results should be important not only for studies on UM pigments but also in many fields of chemistry and material science, where the spectroscopic identification and computational characterization of the elusive S_2_^•–^ radical and, in a broader perspective, also other dichalcogenide radicals, such as Se_2_^•–^, are of relevance.

## 2 Methods and Models

### 2.1 Cluster Models

Cluster models used in this work were cut from the periodic models of UMs, optimized using the pseudopotential density functional theory (DFT) code quantum espresso,^47^ employing the gradient PBE exchange-correlation functional,^48^ as described in detail in the previous paper.^46^ Each periodic model consists of a single cubic unit cell of sodalite lattice with a single S_2_^•–^ radical embedded. Two periodic models chosen correspond to the lowest energy structures of two lattice stoichiometries, namely, (S_2_^•–^)­Na_7_-*sod* (**1**) and (S_2_^•–^)­Na_8_Cl-*sod* (**2**), where *sod* = Si_6_Al_6_O_24_, see Figure 1. Consequently, the cluster models hosting S_2_^•–^ were cut from the optimized periodic structures of type **1** or **2**, and, without any further relaxation, employed in the computation of the spectroscopic quantities of interest. The cluster models used in ref 46 contained only Na and O atoms belonging to the single sodalite cage, encapsulating the S_2_^•–^ ion, with missing O–Si/Al bonds saturated with H atoms, hence forming water molecules. In this work, we employed the cluster models of systematically increasing size, namely: (1) the simplest [Na_4_(S_2_)­(H_2_O)_12_]^3+^ ones, hereafter referred to as small clusters (**1**S, **2**S), where only four Na^+^ ions within the proximity below 3.0 Å to S_2_^•–^ radical are considered, through (2) medium-size clusters, repeated after ref 46, [Na_7_(S_2_)­(H_2_O)_21_]^6+^ (**1**M) and [Na_8_(S_2_)­(H_2_O)_24_]^7+^ (**2**M), up to (3) large clusters, consisting of the whole sodalite cage, with terminal bonds saturated with H atoms, i.e., [Na_7_(S_2_)­Si_6_Al_6_O_60_H_24_]^6–^ (**1**L) and [Na_8_(S_2_)­Si_6_Al_6_O_60_H_24_]^5–^ (**2**L). The clusters of the latter type have been successfully employed in computational studies on red UMs (bearing S_4_ chromophore) by one of the current authors.^49^ Note that using (S_2_^•–^)­Na_7_-*sod* and (S_2_^•–^)­Na_8_Cl-*sod* nomenclature throughout this paper we refer to the stoichiometry of the parent periodic lattice, from which given cluster model was derived, not the actual cluster composition (which were given above). Additional cluster models of S_2_^•–^ defects in NaCl and KCl lattices were constructed using the approach of Stevens et al.^42^ The Cartesian coordinates of all the cluster models used in this study can be found in Supporting Information (SI). The figures were prepared using VESTA^50^ and Gabedit^51^ software.

**Figure 1:**
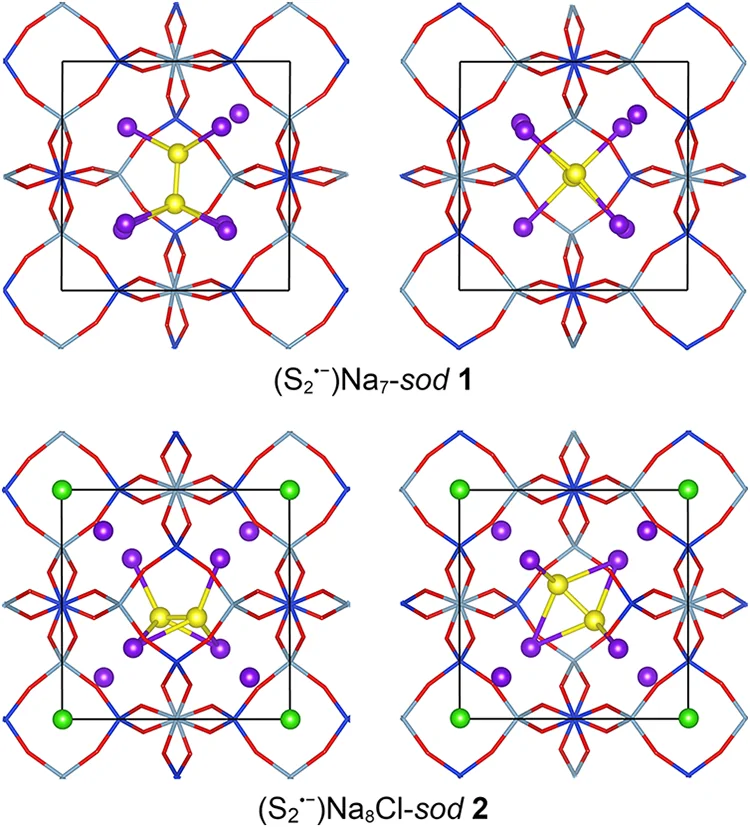
View of S_2_^•–^ radical siting in optimized DFT/PBE periodic models (from ref 46): (S_2_^•–^)­Na_7_-*sod* (**1**), top, and (S_2_^•–^)­Na_8_Cl-*sod* (**2**), bottom, the projections are given along the [001] and [100] axis, at left and right, respectively. Atom colors: S yellow, Na purple, Cl green (balls); Si dark blue, Al light blue, O red (sticks). The optimized structures of (S_2_^•–^)­Na_7_-*sod* and (S_2_^•–^)­Na_8_Cl-*sod* periodic models were used to construct cluster models, of three sizes (small (S), medium (M), and large (L)) for each parent periodic lattice, employed in this work.

### 2.2 DFT Calculations

Single-point, spin-unrestricted Kohn–Sham (UKS) energy calculations were performed with ORCA 5.03 code^52,53^ using Ahlrich triple-ζ valence polarized basis sets (def2-TZVP),^54^ on S atoms minimally augmented with diffuse functions (ma-def2-TZVP).^55^ The following functionals were employed: gradient BP86,^56,57^ hybrid B3LYP,^58^ and long-range corrected CAM-B3LYP,^59^ for the calculations of the *g* tensor, and only the latter one for the time-dependent DFT (TDDFT) calculations of optical excitations in the vertical energy approximation. We found previously that the application of long-range corrected hybrid functionals in TDDFT is usually necessary to avoid likely unphysical low-energy excitations (having the character of charge transfer between the embedded polysulfur chromophore and the lattice) in large cluster models.^49^ In TDDFT calculations, we switched off the Tamm-Dancoff approximation, which is the default in the ORCA code, as it overestimated studied excitation energies by about 0.25 eV (see Table S15).

The EPR *g* tensor was calculated using the coupled-perturbed self-consistent field (CPSCF) approach, as implemented in ORCA,^60^ with the spin–orbit mean-field approximation for the Breit-Pauli spin–orbit coupling (SOC) Hamiltonian.^61,62^ Additional comparative calculations of the *g* tensor, using BP86 and B3LYP functionals, were performed using the ADF package,^63,64^ which enables self-consistent treatment of the SOC^65^ within the zeroth-order regular approximation (ZORA).^66^ The ADF calculations employed spin-restricted open shell Kohn–Sham (ROKS) UKS orbitals, which were expanded using the polarized triple-ζ quality Slater type basis set (TZP),^67^ with additional polarization and diffuse functions for S (AUG/ATZ2P).^68^

### 2.3 CASSCF/CASPT2 Calculations

Single-point energy calculations at the CASPT2 level (with the default 0.25 au IPEA shift^69^ and a 0.1 au imaginary shift) were performed using OpenMolcas v21.06,^70^ based on the active space of 9 electrons in 6 active orbitals (with the S_2_^•–^ σ, π, π*, and σ* character). This is a natural choice of the active space for S_2_^•–^, including all orbitals originating from the sulfur valence 3p subshell, which allows to describe all excited states of interest. The basis set was composed of aug-cc-pV­(T+d)­Z-DK^71,72^ for S and cc-pVDZ-DK^72–75^ for other atoms, and only valence electrons together with the Na 2p outer-core electrons were correlated. To justify this choice, several other basis sets and treatments of the outer-core correlation effects were tested (Tables S5–S8), showing that (i) increasing the basis set on Na atoms to cc-pwCVDZ-DK^75^ (which is, in principle, more suitable for correlating the outer-core electrons) influences the excitation energies only negligibly, as also does correlating the Na 2s electrons; (ii) increasing the basis set on the embedding atoms to triple-ζ quality influences the excitation energies by no more than 0.03 eV at the expense of a much larger computational cost. Scalar-relativistic effects were treated at the second-order Douglas–Kroll–Hess (DKH) level.^76,77^ Nine lowest doublet roots (with the character of S_2_^•– 2^Π_*g*_, ^2^Π_*u*_, ^2^Δ_*u*_, ^2^Σ_*u*_^–^, ^2^Σ_*u*_^+^, ^2^Σ_*g*_^+^; see Section 3.1) were treated in state-average CASSCF, followed by state-specific CASPT2. (Multistate CASPT2 was inspected for selected models, giving essentially identical excitation energies, see Table S10). The *g* tensor for the lowest Kramers doublet was calculated using two methods defined in ref 78, one being first-order degenerate perturbation theory within the isolated Kramers doublet, another one being second-order perturbation theory simultaneously with respect to the SOC and Zeeman operators; both methods as implemented in the state-interaction module (RASSI)^79^ of OpenMolcas. The SOC was treated within the atomic mean-field approximation (AMFI),^61,80,81^ based on the CASSCF/CASPT2 wave functions/energies of the nine doublet states and one quartet state. The angular momentum origin was set to the midpoint of the S–S bond. For isolated S_2_^•–^, comparative energy calculations at the NEVPT2, MRCI+Q, and CCSD­(T) theory levels were performed using Molpro,^82^ employing the same basis set, active space (where applicable), and the DKH scalar-relativistic Hamiltonian.

## 3 Results and Discussion

### 3.1 Electronic States of S_2_^•–^

The electronic ground state of S_2_^•–^ is ^2^Π_*g*_, and the lowest excited states are ^4^Σ_*u*_^–^, ^2^Δ_*u*_, ^2^Π_*u*_, ^2^Σ_*u*_^–^, ^2^Σ_*u*_^+^, and ^2^Σ_*g*_^+^ (see e.g., ref 83). These states originate from different distributions of the 9 electrons in the six active orbitals of the S 3p origin: σ_*g*_ (3p_*z*_) ≡ σ; π_*u*_(3p_*x*,*y*_) ≡ π_*x*,*y*_, π_g_^*^(3p_*x*,*y*_) ≡ π_*x*,y_^*^, σ_u_^*^(3p_*z*_) ≡ σ*. See Figure 2 for contour plots of the S_2_^•–^ orbitals and principal electronic configurations of the considered states. (A detailed derivation of the depicted wave functions can be found in the SI.)

**Figure 2:**
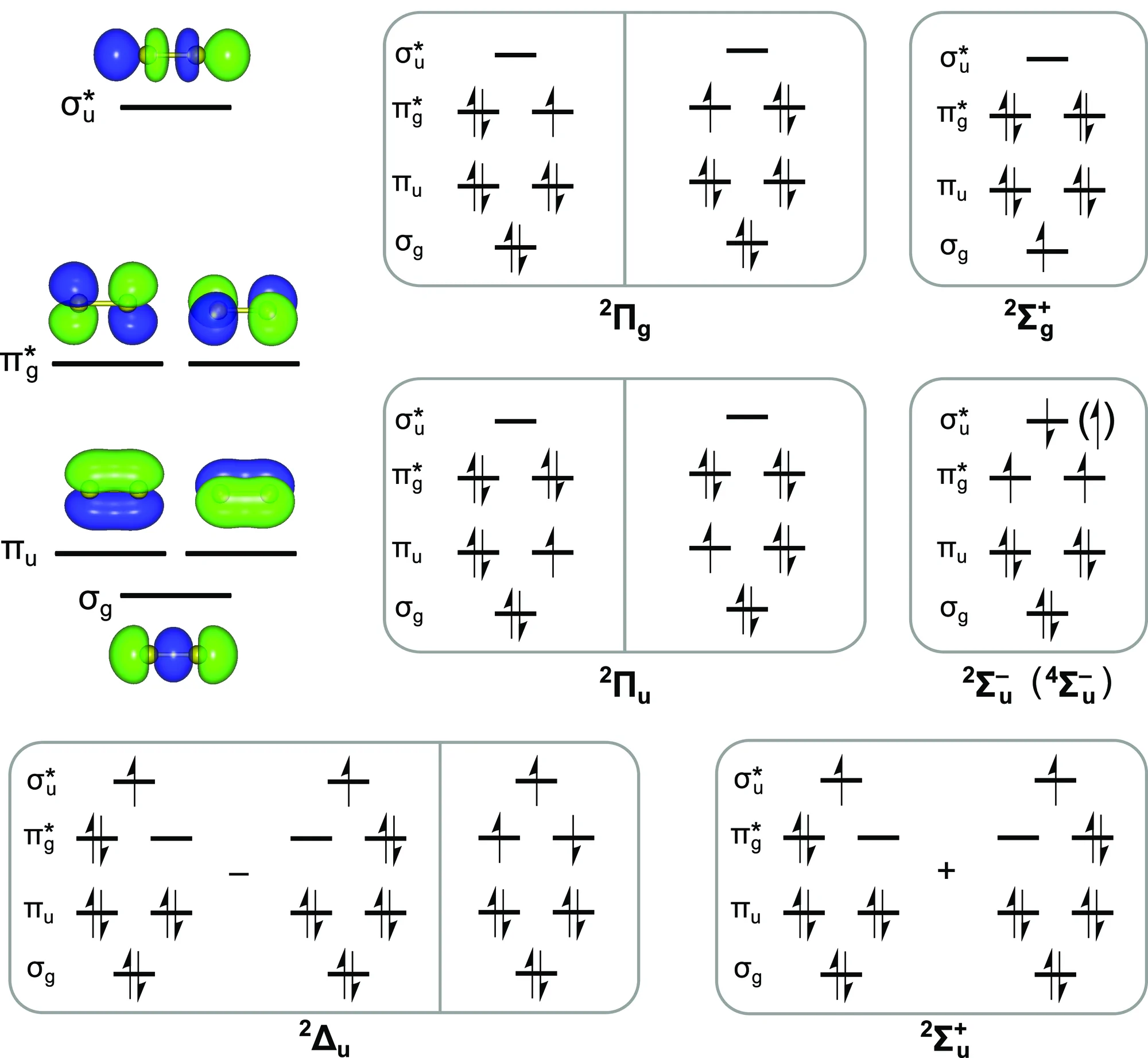
Active orbitals of S_2_^•–^ and schematic electronic configurations for the ground-state ^2^Π_*g*_ and electronically excited states: ^2^Π_*u*_, ^2^Σ_*g*_^+^, ^4^Σ_*u*_^–^, ^2^Σ_*u*_^–^, ^2^Δ_*u*_, ^2^Σ_*u*_^+^. Arrows represent spin couplings, not necessarily individual α/β spins (note that some of these configurations are combinations of several Slater determinants); see details in Table S1.

The electronic states of similar character are also observed for S_2_^•–^ embedded in the considered cluster models. For simplicity, we use the term symbols of isolated S_2_^•–^ also in the UM cluster models to indicate the principal electronic configuration. Obviously, due to the lower symmetry of the UM models, the mapping of their electronic states to those of isolated S_2_^•–^ is only approximate, yet still useful to indicate the predominant character of the electronic states. In the UM models, the energy levels of the Π and Δ character are split into the lower- and higher-energy components, distinguished by adding number (1) or (2) in the superscript, e.g., ^2^Π_*g*_^(1)^ and ^2^Π_*g*_^(2)^. The ground state is Π_*g*_^(1)^ with the unpaired electron, by definition, residing in the π_y_^*^ orbital, and the π_x_^*^ being doubly occupied.

The active orbitals of the S_2_^•–^ chromophore in the considered UM models are depicted in Figure 3 (for the largest considered clusters of the two types). Their similarity to the orbitals of isolated S_2_^•–^ is readily visible, in particular, the quasi-degeneracy of the π_x_^*^ with π_y_^*^ and π_*x*_ with π_*y*_. However, for (S_2_^•–^)­Na_8_Cl-*sod*, one may easily spot some mixing between the σ and one of the π orbitals S_2_^•–^, evidencing the breakage of cylindrical and inversion symmetry by the local environment.

**Figure 3:**
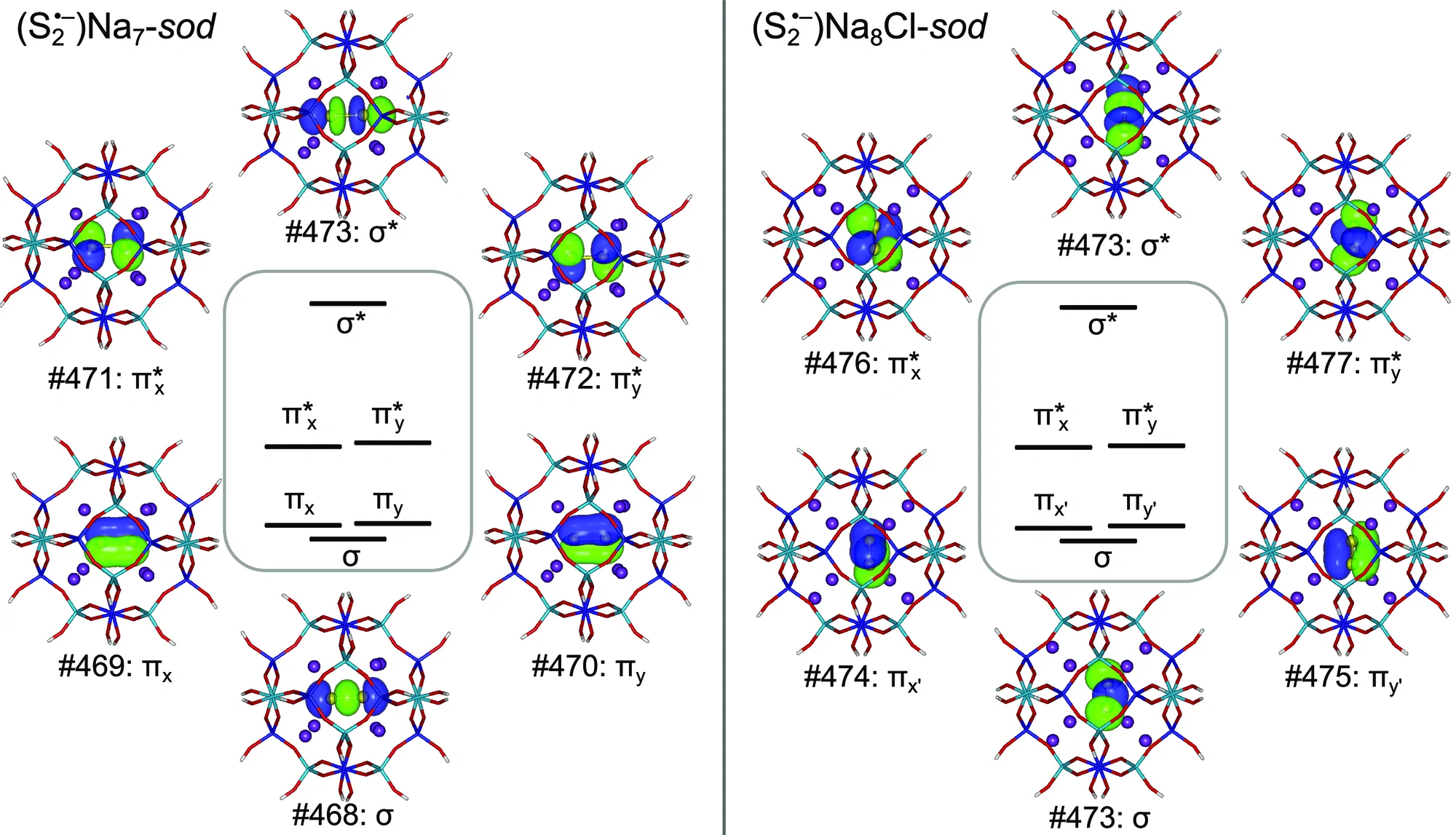
Active orbitals for the large cluster models derived from (S_2_^•–^)­Na_7_-*sod* (left) and (S_2_^•–^)­Na_8_Cl-*sod* (right) lattices. Shown are energy levels and contour plots (±0.04 au) of pseudocanonical orbitals from state-average CASSCF calculations of the nine doublet roots. Note that the lobes of the π_*x*,y_^*^ orbitals are differently oriented for the two models and, for the (S_2_^•–^)­Na_8_Cl-*sod* model, the directions of the π_*x*′,*y*′_ lobes are rotated with respect to the π_*x*,y_^*^ lobes.

It should be stressed that some electronic states of S_2_^•–^ have fundamentally multiconfigurational character (^2^Σ_*u*_^+^, one component of ^2^Δ_*u*_) or their main configuration involves more than one Slater determinant (^2^Σ_*u*_^–^, the second component of ^2^Δ_*u*_); see configurations given in Table S1 and eqs (S1) and (S2). A multiconfigurational method, such as CASSCF/CASPT2, warrants a proper treatment of such states for which static correlation is important. By contrast, the ^2^Π_*g*_ ground state is neither multiconfigurational nor multideterminantal: a given component of it, ^2^Π_*g*_^(1)^ or ^2^Π_*g*_^(2)^, is strongly dominated by a single Slater determinant, with the weight >0.9 (cf Table S2). However, state-specific calculations for the single component give the spin density that is no longer axially symmetric (considering a statistical ensemble of both components would be necessary to restore the axial symmetry, which is roughly equivalent to the usage of fractional occupation numbers in ROKS calculations). Furthermore, the molecular orbitals (MOs) optimized self-consistently for the single component will break the axial symmetry, for example, the π_*x*,y_^*^ orbitals will no longer be equivalent by rotation, and their energy levels will not be degenerate. If the resulting MOs are used to construct the remaining electronic states of S_2_^•–^, the expected degeneracies are broken (e.g., the two components of a formally degenerate state, such as ^2^Π_*g*_ or ^2^Π_*u*_, will have slightly different energies). The problem is unavoidable in the standard UKS procedure and influences the quality of subsequent TDDFT or *g* tensor calculations (see below). However, this issue is elegantly overcome in state-averaged CASSCF calculations, where the MOs are optimized to minimize the average energy of several states, including with equal weights both components for each of the degenerate states (^2^Π_*g*_, ^2^Π_*u*_, and ^2^Δ_*u*_), and thus leading to the MOs that maintain the symmetry of the molecule.

### 3.2 Optical Excitation Energies

Table 1 reports CASPT2 and TDDFT vertical excitation energies and oscillator strengths with respect to the Π_*g*_^(1)^ ground state. The results are given for isolated S_2_^•–^ and green UM models, (S_2_^•–^)­Na_7_-*sod* (**1**) and (S_2_^•–^)­Na_8_Cl-*sod* (**2**). For either model, three increasingly large clusters (S, M, L) are considered to illustrate the convergence of the excitation energies.

**Table 1:** Excitation Energies (eV) and Oscillator Strengths^a^,^b^

				(S_2_^•–^)Na_7_-*sod*				(S_2_^•–^)Na_8_Cl-*sod*			
method	state	S_2_^•–^		**1**S	**1**M	**1**L		**2**S	**2**M	**2**L	
CASPT2	^2^Π_*u*_	3.18	(0.050^c^)	3.23	3.28	3.29	(0.053)	3.32	3.29	3.27	(0.019)
				3.24	3.41	3.43		3.39	3.39	3.37	(0.033)
	^2^Δ_*u*_	2.89^d^	(0.002^e^)	3.98	4.13	4.07		3.89	4.03	4.00	(0.001)
				4.00	4.17	4.13		3.91	4.03	4.00	
	^2^Σ_*u*_^–^	2.70	(0.004)	4.11	4.31	4.26	(0.001)	4.01	4.17	4.14	(0.001)
	^2^Σ_*u*_^+^	3.31	(0.001)	4.41	4.69	4.67		4.38	4.48	4.46	
	^2^Σ_*g*_^+^	4.30		4.09	4.17	4.17		4.24	4.19	4.17	
	^4^Σ_*u*_^–^	2.29		3.22	3.39	3.35		3.14	3.25	3.23	
TDDFT^f^	^2^Π_*u*_	3.01	(0.086)	3.10	3.15	3.16	(0.057)	3.24	3.20	3.17	(0.039)
		3.13^g^		3.20	3.37	3.41		3.32	3.31	3.31	(0.013)
	(π_x_^*^ → σ*)^h^	2.82	(0.031)	4.01	4.22	4.28	(0.004)	3.99	4.04	4.13	(0.003)

a With respect to the ^2^Π_*g*_^(1)^ ground state.

b Oscillator strengths greater than 5 × 10^–4^ are reported in parentheses.

c Oscillator strength for the ^2^Π_*g*_^(1)^ →^2^Π_*u*_^(1)^ transition.

d CASPT2 excitation energies for the ^2^Δ_*u*_^(1)^, ^2^Δ_*u*_^(2)^ differ by 0.015 eV, reported is their average.

e Sum of the oscillator strengths for ^2^Π_*g*_^(1)^ →^2^Δ_*u*_^(1)^,^2^Δ_*u*_^(2)^.

f The CAM-B3LYP functional.

g The splitting of the ^2^Π_*u*_ level in isolated S_2_^•–^ is an artifact (see text).

h The most intense excitation with a prominent π_x_^*^ → σ* character.

In isolated S_2_^•–^, the transitions from ^2^Π_*g*_ to *ungerade* states (^2^Π_*u*_, ^2^Δ_*u*_, ^2^Σ_*u*_^–^, ^2^Σ_*u*_^+^) are dipole-allowed; the transition to ^2^Σ_*g*_^+^ is dipole-forbidden. The table also reports the CASPT2 vertical energy of a spin-forbidden transition to the ^4^Σ_*u*_^–^ state, which appears in a similar energy range. Test CASPT2 calculations with a greater number of states (Table S9) confirm that there are no more excited states within 4 eV (for isolated S_2_^•–^) or 5 eV (for the UM models), at least with the present choice of active space, including six molecular orbitals based on S 3p atomic orbitals. In our discussion, we first focus on the set of CASPT2 energies as being more complete. Note that, by contrast with TDDFT (discussed below), the CASPT2 method warrants a proper treatment of all of the S_2_^•–^ excited states considered in this study.

Going from isolated S_2_^•–^ to UM models, the vertical energy of the ^2^Σ_*g*_^+^ decreases by up to 0.2 eV, that of the ^2^Π_*u*_ increases by up to 0.1–0.2 eV, whereas the energies of the other states increase even more considerably, with the maximum effect of 1 eV for the ^2^Δ_*u*_ and ^2^Σ_*u*_^–^. The states whose energies strongly increase on the embedding have the σ* singly occupied. This orbital is diffuse in isolated S_2_^•–^ and therefore becomes strongly destabilized by the crystal field in UMs, explaining the observed strong dependence. The smallest (S and M) cluster models are apparently too limited to quantitatively describe this effect, as will be detailed below.

We now take an interest in the convergence of the excitation energies with respect to the cluster size (**1**S→**1**M→**1**L, **2**S→**2**M→**2**L). The largest differences in the excitation energies are observed on going from the smallest to the medium-size clusters, especially from **1**S to **1**M, where the observed difference is nearly 0.3 eV for the ^2^Π_*u*_ →^2^Σ_*u*_^+^ excitation and around 0.2 eV for the (higher-energy component of) the ^2^Π_*u*_ →^2^Π_*g*_ excitation. Taking into account all of the CASPT2 excitation energies in Table 1, the mean absolute difference between the small to medium-size clusters is 0.16 eV between **1**S and **1**M for the (S_2_^•–^)­Na_7_-*sod* model and 0.06 eV between **2**S and **2**M models for the (S_2_^•–^)­Na_8_Cl-*sod* model. The effect of further increasing the cluster size is modest and comparable for the two UM models: 0.03 eV between **1**M and **1**L; 0.02 eV between **2**M and **2**L.

The above observations can be rationalized by the difference in the local environment of the S_2_^•–^ radical in models **1** and **2** (cf Figure 1). In the case of **2**, i.e, (S_2_^•–^)­Na_8_Cl-*sod* model, the S_2_^•–^ radical is coordinated by 4 Na^+^ cations in the proximity of less than 3 Å (with [Na_4_(S_2_)]^3+^ moiety having approximately *C*_2*v*_ symmetry), and no other close neighbors, up to the lattice O atoms in the distance of above 3.5 Å, and the next Na^+^ ions are located above 3.8 Å apart. In the case of **1**, i.e., (S_2_^•–^)­Na_7_-*sod* model, there is a gradual increase in the S_2_^•–^ coordination shell, as apart from the nearest neighbor Na^+^ cations within 3 Å radius (here, [Na_4_(S_2_)]^3+^ adduct possesses *C*_2_ symmetry), there is a pair of Na^+^ ions in the distance of 3.1 Å, and then the next O atoms and Na^+^ ions are within the radius of above 3.5 and 3.6 Å, respectively. Therefore, the orbital splitting in (S_2_^•–^)­Na_8_Cl-*sod* model is likely to stem from the strong interaction with the four nearest Na^+^ ions, and to a lesser degree from the long-range interaction with the rest of the lattice, whereas for (S_2_^•–^)­Na_7_-*sod* models, the steady augmentation of the coordination sphere with middle-distance ions leads to the observed slow convergence of the excitation energies with the increasing size of the cluster.

As already mentioned, the vertical excitation energy of the most intense ^2^Π_*g*_ →^2^Π_*u*_ transition is increased in UM models by 0.1–0.2 eV with respect to the isolated S_2_^•–^. Moreover, due to the symmetry lowering, the upper energy level splits into two sublevels, ^2^Π_*u*_^(1)^ and ^2^Π_*u*_^(2)^, separated by ∼ 0.1 eV. In the (S_2_^•–^)­Na_7_-*sod* model, the transition to the lower energy level, ^2^Π_*g*_^(1)^ →^2^Π_*u*_^(1)^ (π_*y*_ → π_y_^*^), accounts for nearly the whole intensity. This resembles the situation in isolated S_2_^•–^, where the ^2^Π_*g*_^(1)^ →^2^Π_*u*_^(2)^ (π_*x*_ → π_y_^*^) transition is symmetry-forbidden. By contrast, in the (S_2_^•–^)­Na_8_Cl-*sod* model, the transitions to both ^2^Π_*u*_^(1)^ and ^2^Π_*u*_^(2)^ energy sublevels gain comparable intensities. Such a result can be rationalized by the shapes of the molecular orbitals involved in these transitions (cf Figure 3). In variance with the case of the isolated S_2_^•–^ or (S_2_^•–^)­Na_7_-*sod* model, the π_*x*,y_^*^ orbitals in the (S_2_^•–^)­Na_8_Cl-*sod* model are no longer oriented consistently with the bonding orbitals, π_*x*′,*y*′_. Moreover, a slight mixing is observed between the σ and π bonding orbitals of the S_2_^•–^ chromophore in (S_2_^•–^)­Na_8_Cl-*sod*, evidencing a greater disturbance of the axial and inversion symmetry than in the case of (S_2_^•–^)­Na_7_-*sod*.

Interestingly, the ^2^Δ_*u*_ and ^2^Σ_*u*_^–^ excited states are lower in energy than ^2^Π_*u*_ in isolated S_2_^•–^, but shift higher in energy (by ∼1 eV) in the UM models. A comparable blue shift due to the embedding of S_2_^•–^ in the cluster is observed for the ^2^Σ_*u*_^+^ and ^4^Σ_*u*_^–^. All of these states involve a single occupation of the σ* orbital, which is diffuse and hence strongly destabilized by the crystal field in UMs (The energies of these states in isolated S_2_^•–^ are also very sensitive to the presence of diffuse functions in the basis set, Table S8). The ^2^Σ_*g*_^+^ state shifts by 0.1–0.2 eV down in energy when comparing isolated S_2_^•–^ with the UM models. The intensities of transitions to all of these states are much smaller than to the bright ^2^Π_*u*_ state. No major differences are observed in the intensities of these transitions between isolated S_2_^•–^ and UM models. The second most intense transition, to the ^2^Σ_*u*_^–^ state, is predicted to be more than an order of magnitude less intense than the ^2^Π_*u*_ state. The transition to the ^2^Σ_*g*_^+^ state, dipole-forbidden in isolated S_2_^•–^, gains some minor intensity (*f*_osc_ = 2 × 10^–4^) in the UM models due to the absence of the inversion center.

We are aware of experimental data only for the most intense ^2^Π_*g*_ →^2^Π_*u*_ transition, with the absorption band maximum 2.9–3.0 eV (428–413 nm) for S_2_^•–^ in neon matrix^23^ or around 3.1 eV (400 nm) in green UM.^45^ The corresponding CASPT2 results are 3.2 eV for isolated S_2_^•–^ and 3.3–3.4 eV for the UM cluster models (as Table 1 shows, the excitation energy converges rapidly with the model size). The CASPT2 excitation energies are thus higher by 0.2–0.3 eV than the experimental band maxima. While not perfect, we consider this a rather satisfactory agreement with the experiment, especially given the breadth of the experimental bands and the absence of vibronic effects in the present calculations. The influence of vibronic effects on S_2_^•–^ emission was shown to be rather small (shifting the transitions by about 0.1 eV) by Colinet et al.;^38^ hence, we do not expect the strong impact on absorption.

The excitation energies for isolated S_2_^•–^ were also comparatively calculated at the NEVPT2, MRCI+Q, and CCSD­(T) theory levels (Table S11). Concerning the most relevant ^2^Π_*g*_ →^2^ Π_*u*_ excitation, the CASPT2 and NEVPT2 energies agree well, but MRCI+Q and CCSD­(T) energies are slightly lower, around 3.1 eV, and closer to the experimental band position. A similar slight lowering of the ^2^Π_*g*_ →^2^ Π_*u*_ excitation energy is also observed in the CASPT2 method when changing the IPEA shift parameter from the default value of 0.25 au to zero; however, the default choice gives a better overall match with the MRCI+Q excitation energies (Table S11).

We further note that the ^2^Π_*g*_ →^2^Π_*u*_ vertical excitation energy is sensitive to the S–S bond distance, with the slope of about −0.05 eV/pm (Figure S1). All of the excitation energies discussed so far were calculated based on the geometries optimized using the PBE functional. The PBE distance in isolated S_2_^•–^ is 2.020 Å and is essentially identical with the distances obtained at the B3LYP and CCSD­(T) theory levels (Table S12); we therefore deem this distance to be reliable. (The equilibrium distance of 2.06 Å, reported in the earlier study by Arieli et al.,^84^ seems to be unrealistically long. We suspect it might arise from a typographic error in the mentioned paper: note that they reported the same bond length for S_3_^•–^.) For comparison, the value of 1.996 Å was obtained recently from DFT calculations with the hybrid PBE0 functional for S_2_^•–^.^2^ Sticking to the PBE geometries, the S–S distance in the considered UM models is shorter (by 0.003 to 0.007 Å) than in isolated S_2_^•–^. This slight contraction of the bond agrees with the earlier discussed increase in the excitation energy in the UM models. However, the observed magnitude of the energy increase (0.1–0.2 eV) is several times greater than it would be anticipated solely from the S–S bond contraction.

For the UM models, the TDDFT and CASPT2 excitation energies agree to within 0.15 eV. However, the TDDFT fails to predict degeneracy between two components of the ^2^Π_*u*_ state in isolated S_2_^•–^, which is an artifact arising from breaking of the cylindrical symmetry in the Slater determinant describing the ^2^Π_*g*_^(1)^, a single component of the doubly degenerate ground state. Despite this problem in isolated S_2_^•–^, the splitting between the lower and upper ^2^Π_*u*_ levels in UM models is described rather similarly by TDDFT as by CASPT2.

The results of TDDFT (CAM-B3LYP) calculations are summarized in the bottom part of Table 1 and in Figure 4, where, for clarity, we characterized selected excitations employing the Natural Transition Orbitals (NTOs) picture.^85^ Discussing the TDDFT results, we focus on the most intense transitions ^2^Π_*g*_ →^2^Π_*u*_, which are described well as single-electron excitations with respect to the ground-state determinant. For this transition in a given model, the TDDFT and CASPT2 results agree to within 0.15 eV. However, the TDDFT calculations fail to predict degeneracy between two components of the ^2^Π_*u*_ state in isolated S_2_^•–^, which is an artifact arising from breaking of the cylindrical symmetry in the Slater determinant describing the ^2^Π_*g*_^(1)^, a single component of the doubly degenerate ground state (see comment in Section 3.1). Despite this problem in isolated S_2_^•–^, the splitting between the lower and upper ^2^Π_*u*_ levels in UM models is described rather similarly by TDDFT as by CASPT2. The most intense component of the ^2^Π_*g*_ →^2^Π_*u*_ excitation, namely, ^2^Π_*g*_^(1)^ →^2^Π_*u*_^(1)^, which is due to the π_*y*_ → π_y_^*^ transition, is predicted in both considered UM models at about 3.2 eV (390 nm), therefore in a good agreement with the experimental data.^1,16,27^ Analogously to the CASPT2 results discussed above, in the (S_2_^•–^)­Na_8_Cl-*sod* model, another intense transition is predicted at 3.3 eV (375 nm), stemming from the (π_*u*_)_*x*_ → (π_g_^*^)_*y*_ excitation (^2^Π_*g*_^(1)^ →^2^Π_*u*_^(2)^). Although the latter one would be forbidden in an isolated S_2_^•–^ radical (*D*_∞*h*_ symmetry), it is relatively intense in the (S_2_^•–^)­Na_8_Cl-*sod* model due to the symmetry lowering. In particular, the shape of the (π_*u*_)_*y*_ orbital is strongly distorted due to interaction with Na^+^ ions coordinating S_2_^•–^ by side; as can be seen in Figure 4, the nodal plane of the aforementioned (π_*u*_)_*y*_ orbital is curved, and the orbital is less extent at the concave side of this surface. In the (S_2_^•–^)­Na_7_-*sod* model, where S_2_^•–^ is coordinated by Na^+^ ions in a manner preserving the axial symmetry of [Na_4_(S_2_)]^3+^ adduct, thus no perturbation perpendicular to the S–S bond is present, the (π_*u*_)_*y*_ → (π_g_^*^)_*x*_ transition remains virtually forbidden (*f*_osc_ ∼ 10^–6^). The latter excitation can possibly contribute to the broadening and tailing toward higher energies of the main band at 400 nm.

**Figure 4:**
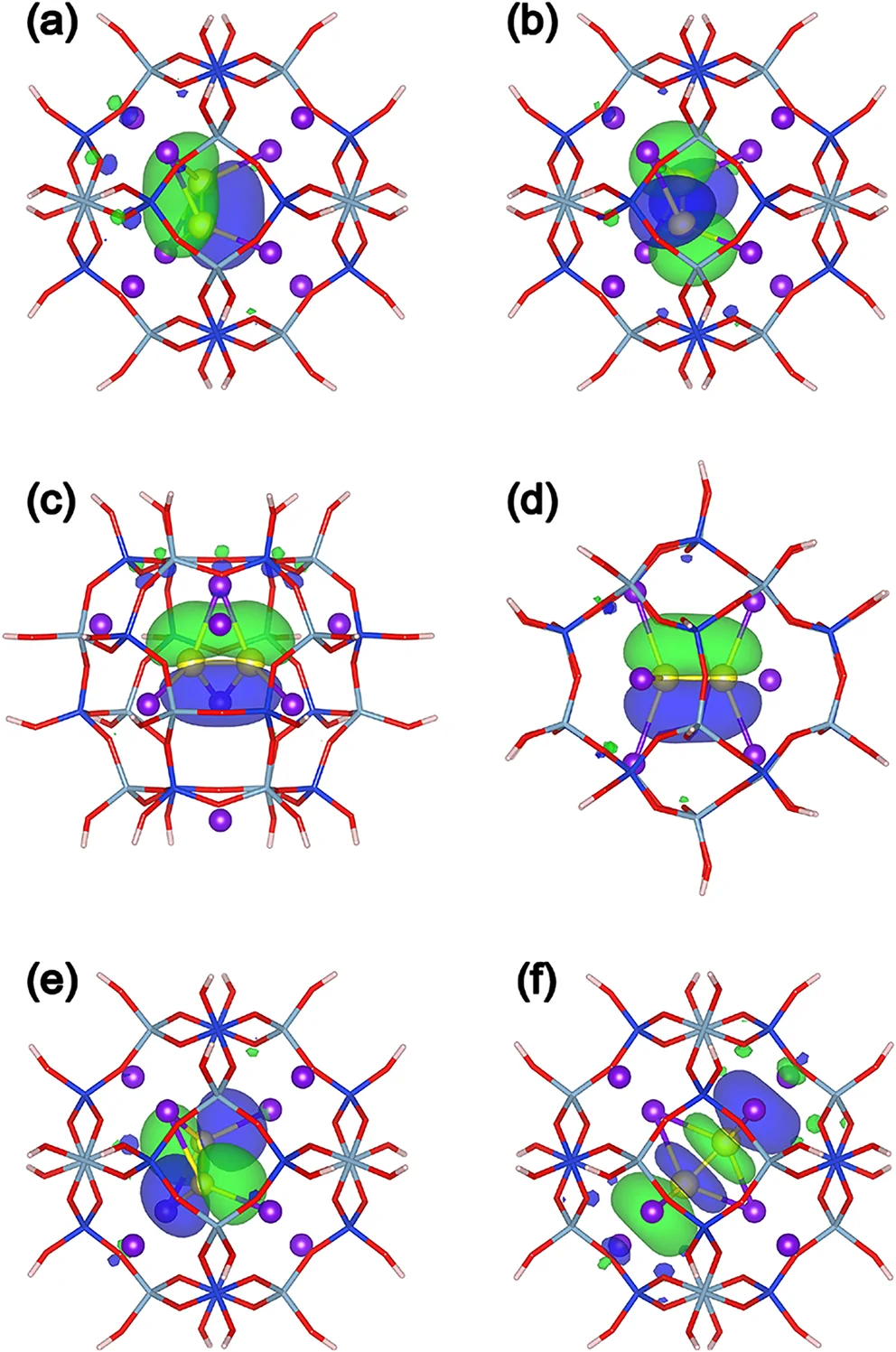
NTOs for selected TDDFT electron excitations. Top panel: (a) initial and (b) final NTOs corresponding to (π_*u*_)_*y*_ → (π_g_^*^)_*y*_ (SOMO) excitation at 3.17 eV in the **2**L model; middle panel: the comparison of initial states in forbidden (π_*u*_)_*x*_ → (π_g_^*^)_*y*_ transition, in (c) the **2**L model at 3.31, and (d) in the **1**L model, at 3.41 eV—the final states of these transitions, not shown, look like (b) NTO (i.e, SOMO); bottom panel: (e) initial, and (f) final NTOs corresponding to the (π_u_^*^)_*x*_ → (σ*)_*z*_ excitation at 4.13 eV in the **2**L model.

In the bottom of Table 1, we also included the energies of the most intense excitations with predominantly π_x_^*^ → σ* character, which occur at 2.8 eV for S_2_^•–^ and above 4.0 eV for the cluster models. Based on the character of the involved NTOs and the energy range, these excitations should be associated with the ^2^Δ_*u*_ or ^2^Σ_*u*_^–^ states of S_2_^–•^. However, the detailed assignment of these states is problematic in the frame of the TDDFT calculations. Note that proper description of the ^2^Δ_*u*_ and ^2^Σ_*u*_^–^ states in S_2_^•–^ requires participation of Slater determinants being double excitations with respect to the ground-state determinant, and hence, the TDDFT treatment is not expected to be accurate for the π_x_^*^ → σ* excitations. In particular, it does not allow to easily distinguish between the ^2^Δ_*u*_ and ^2^Σ_*u*_^–^ state due to the lack of doubly excited determinants. Nonetheless, the energy of the π_x_^*^ → σ* excitation in TDDFT is within 0.1 eV identical to the average energy of the ^2^Δ_*u*_ and ^2^Σ_*u*_^–^ states obtained from CASPT2 calculations.

In the preliminary report on S_2_^•–^ sites in UM in ref 46, one of the current authors reported a strong discrepancy between the excitation energies (and *g*_iso_ as well) calculated for (S_2_^•–^)­Na_7_-*sod* and (S_2_^•–^)­Na_8_Cl-*sod* models, the latter showing values too high (4.29 eV or 289 nm) with respect to the experimental ones. A more careful inspection, including the stability analysis of the SCF solution, showed that this was the result of accidentally converging the (S_2_^•–^)­Na_8_Cl-*sod* wave function to an excited state with a different π_g_^*^ component being singly occupied than in the ground state.

### 3.3 Electron Paramagnetic *g* Tensor

#### 3.3.1 Comparison of Theoretical Approaches

The methods of *g* tensor prediction can be generally divided into two classes, namely, the first- and second-order approaches, depending on whether spin–orbit coupling (SOC) is treated variationally or perturbatively.^86,87^ The perturbative treatment of the SOC is not expected to be valid for an orbitally degenerate ground state, like here for S_2_^•–^, but since the earlier studies most relevant for our present work employed such an approach (for isolated S_2_^•–^ radical,^84^ and embedded in the environment of point charges, mimicking the lattices of sodalite and cancrinite minerals^88^) or both approaches (for S_2_^•–^ defects in alkali halides),^42^ we also feel obliged to systematically compare the first- and second-order approaches for S_2_^•–^ in UM models. At the CASPT2 level, we thus consider either the first-order degenerate perturbation theory treatment of the Zeeman term within the isolated ground-state Kramers doublet or the sum-overstates approach, in which the SOC and the Zeeman term are treated simultaneously using second-order perturbation theory; both of these methods are used as formulated by Vancoillie, Malmqvist, and Pierloot.^78^ At the DFT level, the second-order approach is far more common in computational packages (e.g., ORCA, Gaussian) as it can be conveniently implemented within the nonrelativistic Hamiltonian framework using the linear response formalism (coupled-perturbed SCF); we use the formulation and implementation by Neese.^60,62^ As an alternative, the first-order approach is implemented, for example, in the ADF code, where self-consistent SOC treatment was originally developed for spin-restricted (ROKS) calculations by van Lenthe et al.^65^ However, recent versions of ADF also support spin-unrestricted (UKS) self-consistent SOC calculation of the g-tensors within the collinear approximation^89^

Table 2 reports the principal *g* values calculated using first- and second-order approaches at CASPT2 and several DFT levels. The results are given for isolated S_2_^•–^ and the same radical embedded in the cluster models of green UM. In all cases, the calculated *g* tensor is correctly predicted to be anisotropic. Importantly, the largest value (*g*_*z*_) corresponds to the S–S bond direction. Wherever the two smaller principal values differ, the smallest one (*g*_*y*_) corresponds to the direction of the lobes of the π_y_^*^ (SOMO) in the ^2^Π_*g*_^(1)^ ground state.

**Table 2:** Calculated EPR *g* Values for Isolated S_2_^•–^ and UM Models

				(S_2_^•–^)Na_7_-*sod*			(S_2_^•–^)Na_8_Cl-*sod*		
approach^a^	theory level	*g*_*i*_	S_2_^•–^	**1**S	**1**M	**1**L	**2**S	**2**M	**2**L
1st order	CASPT2	*g*_*x*_	0.000	0.262	1.980	1.991	1.916	1.814	1.786
		*g*_*y*_	0.000	0.261	1.960	1.970	1.898	1.799	1.772
		*g*_*z*_	4.002	3.960	2.419	2.368	2.643	2.879	2.930
	BP86/ROKS^b^	*g*_*x*_	0.000	0.048	1.031	1.163	0.684	0.519	0.485
		*g*_*y*_	0.000	0.048	1.026	1.157	0.682	0.518	0.484
		*g*_*z*_	4.004	3.948	3.659	3.576	3.836	3.864	3.860
	BP86/UKS^b^	*g*_*x*_	2.004	2.004	2.020	2.021	2.016	2.013	2.012
		*g*_*y*_	2.004	1.978	1.993	1.994	1.983	1.980	1.980
		*g*_*z*_	4.004	3.937	2.291	2.393	2.269	2.436	2.442
	B3LYP/ROKS^b^	*g*_*x*_	0.000	0.047	1.011	1.130	0.678	0.527	^e^
		*g*_*y*_	0.000	0.047	1.006	1.124	0.676	0.526	^e^
		*g*_*z*_	4.004	3.953	3.661	3.588	3.853	3.891	^e^
2nd order	CASPT2	*g*_*x*_	^c^	2.029	2.028	2.028	2.028	2.029	2.029
		*g*_*y*_	^c^	2.002	2.002	2.002	2.002	2.002	2.002
		*g*_*z*_	^c^	17.184	2.432	2.377	2.683	2.986	3.060
	BP86^d^	*g*_*x*_	2.030	2.030	2.030	2.030	2.031	2.031	2.031
		*g*_*y*_	2.002	2.002	2.002	2.002	2.002	2.002	2.002
		*g*_*z*_	2.712	2.704	2.290	2.266	2.386	2.433	2.435
	B3LYP^d^	*g*_*x*_	2.033	2.034	2.033	2.033	2.034	2.034	2.034
		*g*_*y*_	2.002	2.002	2.002	2.002	2.002	2.002	2.002
		*g*_*z*_	3.137	3.055	2.333	2.302	2.458	2.522	2.523
	CAM-B3LYP^d^	*g*_*x*_	2.037	2.034	2.036	2.035	2.037	2.037	2.037
		*g*_*y*_	2.002	2.002	2.002	2.002	2.002	2.002	2.002
		*g*_*z*_	4.008	3.669	2.374	2.336	2.540	2.632	2.634

a Approach used to calculate the *g* tensor, depending on the treatment of the SOC (see text).

b ADF.

c This is not feasible for a degenerate state.

d ORCA, UKS calculations.

e Calculations did not converge.

The anisotropy of the *g* tensor is reminiscent of the strong SOC within the orbitally degenerate ^2^Π_*g*_ ground state of isolated S_2_^•–^, yielding the lowest Kramers doublet (^2^Π_3/2_) with *g*_*z*_ ≡ *g*_∥_ ≈ 4 and *g*_*x*_ = *g*_*y*_ ≡ *g*_⊥_ ≈ 0.^90^ Such *g* values for isolated S_2_^•–^ are indeed obtained from the first-order approach at the CASPT2 and DFT/ROKS theory levels, but not at the DFT/UKS level (Table 2). The latter gives instead *g*_*x*_ and *g*_*y*_ ≈ 2, very far from the expected (zero) values. In fact, the present BP86 *g* tensor of isolated S_2_^•–^ computed within the second-order approach (2.030, 2.002, 2.712) is very similar to that reported by Arieli et al.^84^ (2.034, 2.002, 2.858) using the same approach and the choice of functional. The *g*_*z*_ value varies with the functional (and, interestingly, with the CAM-B3LYP functional, it approaches the expected value of 4). However, the *g*_*x*_ and *g*_*y*_ values of about 2 are nearly functional-independent and clearly incorrect. The failure of the second-order approach to reproduce the *g* tensor of isolated S_2_^•–^ is not an issue of a specific exchange-correlation functional, but rather a general problem with the linear response procedure based on UKS orbitals optimized self-consistently for one component of the degenerate ^2^Π_*g*_ state. The orbitals optimal for one component are far from optimal for the other, leading to artificial breaking of the degeneracy between the two components, making it impossible to correctly describe the strong SOC effect within the orbitally degenerate ^2^Π_*g*_ ground state. (A related problem with the degeneracy breaking in isolated S_2_^•–^ was manifested already in the TDDFT calculations, discussed in the previous section, where the (π_*u*_)_*y*_ → (π_g_^*^)_*y*_ and (π_*u*_)_*x*_ → (π_g_^*^)_*y*_ excitations artificially differed in energy by 0.1 eV, although they are supposed to describe degenerate components of the ^2^Π_*g*_ →^2^Π_*u*_ transition; cf Table 1.) It is due to this artificial degeneracy breaking in the UKS calculations that the perturbative SOC treatment remains formally valid (although it does not produce the correct *g* tensor either) and hence the problem may easily go unnoticed, as presumably happened in ref 84. Similar problems with the second-order approach persist in UM models even though they no longer have exact orbital degeneracy (see below).

We now focus on the first-order CASPT2 results (top part of Table 2) because this approach appears to be best suited to simultaneously address the strong SOC and near-degeneracy effects crucial for the *g* tensor prediction. As mentioned above, the *g* values of isolated S_2_^•–^ are reproduced correctly. Upon embedding of S_2_^•–^ in the sodalite cages of UMs, the orbital degeneracy of the ^2^Π_*g*_ state is lifted, reducing the *g* tensor anisotropy. However, the CASPT2 still first-order calculations predict *g*_*x*_, *g*_*y*_ < *g*_*e*_ and *g*_*z*_ > *g*_*e*_ (where *g*_*e*_ = 2.002 is the free electron *g* factor). The calculated *g* values depend on the model and cluster size. Going from **1**S to **1**M, the variations of the *g* values are enormous, but even comparing the medium-size (**1**M, **2**M) and large clusters (**1**L, **2**L), the *g* values still differ by 0.05 (*g*_*z*_) or 0.03 (*g*_*x*_). While the numeric *g* values may not be fully converged with respect to the cluster size (S → M → L), they are sufficiently converged to see the difference between the models of type **1** and **2**. In the (S_2_^•–^)­Na_7_-*sod* model (**1**), the *g*_*x*_ and *g*_*y*_ values are slightly below the *g*_*e*_ and the *g*_*z*_ value is ∼2.4. In the (S_2_^•–^)­Na_8_Cl-*sod* model (**2**), the *g*_*x*_ and *g*_*y*_ values are ∼ 1.8, thus significantly below the *g*_*e*_, and the *g*_*z*_ value is as high as ∼2.9.

The large sensitivity of the *g* tensor to the local environment of S_2_^•–^ in different models should be contrasted with relatively insensitive optical excitation energies (see previous section). The reason for this different behavior is, obviously, the sensitivity of the *g* tensor to the splitting of the two ^2^Π_*g*_ levels, i.e., a relatively small energy difference between the ^2^Π_*g*_^(2)^ and ^2^Π_*g*_^(1)^ states, which is strongly dependent on the local environment in different clusters (Table S13). The splitting calculated without considering the SOC varies from zero in isolated S_2_^•–^ or only 0.01 eV in **1**S, through 0.1 eV in **2**S, **2**M and **2**L, up to 0.2 eV in **1**M and **1**L. If the SOC is added to the Hamiltonian, the splitting between the two lowest Kramers doublets for S_2_^•–^ and **1**S increases to 0.05 eV, whereas in the other cases the splitting changes negligibly (cf Table S13). As might be expected, **1**S with the smallest splitting gives the *g* tensor very similar to that of isolated S_2_^•–^: *g*_*z*_ = 3.96 and *g*_*x*_ ≈ *g*_*y*_ = 0.26. We note in passing that the discussed splitting of the ^2^Π_*g*_ states is well approximated by the orbital energy differences between the π_y_^*^ and π_x_^*^ orbitals at the CASSCF level (pseudocanonical orbitals), also given in Table S13.

The first-order DFT calculations of the *g* tensor were also performed, but the results are not particularly encouraging (cf Table 2). The ROKS calculations correctly reproduce *g* tensor components for gaseous S_2_^•–^, but the values obtained for the radical embedded in UM models remain too close to the gas phase ones, with *g*_*x*,*y*_ components being too small (0.05–1.16), and *g*_*z*_ being too large (>3.5), with respect to the first-order CASPT2 data. No principal values close to 2 appear in these ROKS first-order calculations for any of the considered UM models. The UKS calculations give a better agreement with the CASPT2 results for the embedded species, but they incorrectly yield nonzero *g*_*x*,*y*_ components for isolated S_2_^•–^. It is also interesting to notice that the UKS calculations for UM models predict a much larger difference between the *g*_*x*_ and *g*_*y*_ values (0.02–0.03) than the analogous ROKS calculations (predicting *g*_*x*_ and *g*_*y*_ values identical to within 0.006) or the corresponding CASPT2 calculations.

The second-order CASPT2 approach cannot be applied to isolated S_2_^•–^ due to the exact orbital degeneracy. Although it can be applied to UM models, where the degeneracy is lifted, this approach is not expected^78^ to correctly describe cases with strong SOC and therefore we do not consider the results reliable. Accordingly, the predicted *g*_*x*_ ≈ 2.03 and *g*_*y*_ ≈ *g*_*e*_ values (which are nearly independent of the model) and the *g*_*z*_ values (which is more sensitive to the model) are very different from the corresponding first-order CASPT2 results.

The same holds true for the second-order UKS calculations, which yield *g*_*x*_ = 2.03–2.04 and *g*_*y*_ ≈ *g*_*e*_ independently of the model, cluster size, and the choice of functional. The variation of the *g*_*z*_ value with the choice of functional is usually within 0.2, except for the isolated S_2_^•–^ and the small (S_2_^•–^)­Na_7_-*sod* cluster (**1**S), for which the *g*_*z*_ value varies more significantly. Overall, we consider the *g* value from the second-order UKS calculations unreliable due to their dissimilarity with the first-order CASPT2 results and, in particular, the lack of *g* values below 2 (which are clearly suggested by the experimental data for UMs, see below). These shortcomings are rooted in the aforementioned problem with the linear response UKS calculations to correctly account for the quasi-degeneracy of the ^2^Π_*g*_^(1)^ and ^2^Π_*g*_^(2)^ states with the set of orbitals optimized for only one of these states, resulting in a highly deficient treatment of the SOC. It may also be interesting to note in passing that the splitting between the π_x_^*^ and π_y_^*^ UKS orbitals is strongly functional dependent and can hardly be equated to the splitting between the ^2^Π_*g*_^(1)^ and ^2^Π_*g*_^(2)^ energy levels, especially for CAM-B3LYP (cf Table S13). The latter stems from the fact that TDDFT excitation energy, apart from the difference between ground-state occupied and virtual orbital energies, contains the terms describing excited electron–hole interaction, which is particularly large for long-range corrected functionals.^91^

We further note that for a given level of *g* tensor treatment (1st order/ROKS and second-order/UKS), the DFT results are not very sensitive to the choice of exchange-correlation functional, gradient or hybrid. This is to be contrasted with a much greater sensitivity reported for S_2_^•–^ defects in alkali halides by Stevens et al., who claimed major discrepancies between the results from pure and hybrid functionals.^42^ Based on our results for S_2_^•–^ in UM models, we believe that the discrepancies observed in ref 42 should be rather attributed to the usage of different approaches in the *g* tensor calculations, as Stevens et al. in most cases compared the first-order ROKS data for pure functionals with the second-order/UKS results for hybrid ones. Additionally, the *g*_*z*_ value is quite sensitive to the basis set, thus the reported poorer performance of hybrid functionals in ref 42 could be at least partially explained by the usage of somewhat insufficient Gaussian basis sets (Table S14).

#### 3.3.2 Comparison with Experiment

Table 3 shows experimental and calculated *g* values for S_2_^•–^ in green UM^45^ and selected alkali halides.^18,19^ The calculated results are limited to the first-order CASPT2 approach, which, as discussed in the previous section, appears to be the most appropriate theoretical approach to reliably describe the SOC and quasi-degeneracy effects relevant for the *g* tensor determination. The calculated results are given for the most extensive cluster models (**1**L, **2**L) representing the two sitings of S_2_^•–^ in UM (cf Figure 1) and the cluster models representing S_2_^•–^ defects in NaCl and KCl ionic lattices, following the study of Stevens et al.^42^ In addition to the principal values (*g*_*x*_, *g*_*y*_, *g*_*z*_), the table also displays the isotropic value of the *g* tensor, *g*_iso_ = (*g*_*x*_ + *g*_*y*_ + *g*_*z*_)/3, as well as its axiality Δ*g* = *g*_*z*_ – (*g*_*x*_ + *g*_*y*_)/2 and rhombicity *δg* = *g*_*x*_ – *g*_*y*_.

**Table 3:** *g* Values of S_2_^•–^ in Various Media

sample	method	*g*_*x*_	*g*_*y*_	*g*_*z*_	*g*_iso_ ^a^	Δ*g* ^b^	*δg* ^c^
UM	exptl^d^	2.03(4)	1.85(6)	2.69(6)	2.19(5)	0.75	0.18
	calcd^e^ **1**L	1.991	1.970	2.368	2.110	0.388	0.021
	calcd^e^ **2**L	1.786	1.772	2.930	2.163	1.151	0.014
NaCl	exptl^f^	2.011	1.986	2.253	2.083	0.254	0.025
	calcd^e^	2.009	1.985	2.264	2.086	0.267	0.024
KCl	exptl^g^	0.950	0.948	3.430	1.776	2.481	0.002
	calcd^e^	1.798	1.784	2.890	2.157	1.099	0.004
KBr	exptl^g^	0.843	0.839	3.504	1.729	2.663	0.004
KI	exptl^g^	1.637	1.625	3.063	2.108	1.432	0.012
RbI	exptl^g^	1.297	1.290	3.360	1.982	2.066	0.007
RbBr	exptl^g^	1.757	1.745	2.894	2.132	1.143	0.012

a Isotropic value: (*g*_*x*_ + *g*_*y*_ + *g*_*z*_)/3.

b Axiality, Δ*g* = *g*_*z*_ – (*g*_*x*_ + *g*_*y*_)/2.

c Rhombicity, *δg* = *g*_*x*_ – *g*_*y*_.

d Ref 45.

e This work, CASPT2 method, first-order approach.

f Ref 92.

g Ref 18.

As shown in Table 3, the CASPT2 calculations for UM models predict a strongly anisotropic *g* tensor with the *g*_*x*_ ≈ *g*_*y*_ < 2 and the *g*_*z*_ > 2.3. Such approximately tetragonal symmetry of the *g* tensor was reported in the majority of EPR experiments for S_2_^•–^ defects in alkali halides (see representative data in Table 3, more in ref 42 and refs therein). The large differences between the *g* tensors for models **1**L and **2**L are due to the sensitivity of π_y_^*^ – π_x_^*^ orbital splitting to the local environment (i.e., Na^+^ coordination), which implies the possible presence of several nonequivalent S_2_^•–^ paramagnetic centers in a single UM sample. Such results qualitatively agree with the wide range of *g* values observed experimentally for S_2_^•–^ defects in alkali halides, depending on the chemical environment. The strong *g* tensor anisotropy and the large *g*_*z*_ values presently calculated for both UM models agree, at least qualitatively, with the experimental results of Raulin et al.,^45^ allowing us to reject some earlier assignments of the isotropic EPR signals to S_2_^•–^ in UM.^16,43^ However, the numeric *g* values calculated for either UM model do not match the corresponding experimental values and, in particular, the proximity of the *g*_*x*_ and *g*_*y*_ values calculated for a given model is inconsistent with the data from ref 45, where the reported *g*_*x*_ and *g*_*y*_ values differ by as much as 0.18.

The difference between the *g*_*x*_ and *g*_*y*_ values is the rhombicity of the *g* tensor (*δg*), which is listed for convenience in the last column of Table 3. The value *δg* = 0.18 from the experimental study in ref 45 appears to be unusually high for a trapped S_2_^•–^ radical based on comparison with other experimentally characterized systems (cf Table 3). Even though the *g* values for S_2_^•–^ in alkali halides show large variations depending on the chemical environment, the rhombicities never exceed 0.030 and are usually within 0.015. To gauge the accuracy of our computational protocol, we have tested it on two cases of S_2_^•–^ doped alkali halides previously studied by Stevens et al.:^42^ NaCl, showing the largest rhombicity (*δg* = 0.025), and KCl, showing the smallest rhombicity (*δg* = 0.002), among the alkali halides are considered in ref 42.^93^ In both cases the experimental rhombicities *δg* are reproduced to within 0.002 (even if for KCl the isotropic value *g*_iso_ is overestimated and the axiality Δ*g* is admittedly underestimated with respect to the experiment). Thus, the computational protocol used here is unlikely to underestimate the rhomobicity of the *g* tensor in UM models by about an order of magnitude, as it would seem from a naive comparison of the calculated results (*δg* = 0.014 or 0.021, for models **1**L and **2**L, respectively) with the experimental value (*δg* = 0.18) based on ref 45. On the contrary, while we do not have ultimate trust in the calculated *g* values (in particular, not in *g*_iso_ and Δ*g* values, which are model-sensitive), it is the unusually large rhombicity of the experimental *g* tensor that appears suspicious.

Some insights could also be obtained from the phenomenological theory of the *g* tensor given by Känzig, Cohen, and Zeller (KCZ),^94,95^ with the following approximate formulas:

1 $$g_{x,y}=g_{e}\cos\theta +\frac{\lambda }{E}\left\{\cos2\alpha \pm \left(1-\sin\theta \right)\right\}$$

2 $$g_{z}=g_{e}+2l\sin\theta$$

3 where⁢tan⁡θ=λ/Δ

The parameter λ is the strength of the SOC, *l* is the effective angular momentum on the S–S axis, Δ is the splitting between the π_x_^*^ and π_y_^*^ orbitals, and *E* is ^2^Π_*g*_ →^2^Σ_*g*_^+^ excitation energy. In isolated S_2_^•–^ one has *l* = 1, Δ = 0 and $\theta =\frac{\pi }{2}$ . In Table 4, the parameters λ/Δ, *l*, and λ/*E* were determined based on eqs 1–3 from the experimental *g* values for S_2_^•–^ in different environments.

**Table 4:** Parameters of KCZ Model Derived from Experimental *g* Values

system	λ/Δ	*l*	λ/*E*
S_2_^•–^ in UM^a^	0.50	0.77	0.16
NaCl:S_2_^•–^ ^b^	0.13	0.97	0.014
	0.14	0.90	0.014
KCl:S_2_^•–^ ^c^	1.87	0.81	0.008
KBr:S_2_^•–^ ^c^	2.19	0.82	0.022
KI:S_2_^•–^ ^c^	0.73	0.90	0.015
RbI:S_2_^•–^ ^c^	1.20	0.88	0.015
RbBr:S_2_^•–^ ^c^	0.57	0.90	0.012

a Based on experimental data from ref 45.

b Based on experimental data from refs^19^ and^92^.

c Based on experimental data from ref 18.

The parameter λ/Δ varies from 0.13 to 2.2, and the value obtained for UM is thus typical. The parameter *l* varies from 0.77 to nearly 1, and the lowest value, clearly distinct from others, is that obtained for UM. Finally, the λ/*E* ratio obtained for S_2_^•–^ in UM (0.16) is nearly an order of magnitude larger than for S_2_^•–^ in any other environment (0.0085–0.0222). As there is no reason for λ (i.e., the SOC strength) to vary so significantly for the same S_2_^•–^ radical in different environments, the only possibility to explain it would be an outstandingly small *E* value in the denominator. However, we recall from previous discussion that in UM models the energy of ^2^Π_*g*_ →^2^Σ_*g*_^+^ excitation, usually associated with the *E* parameter, is quite large (>4 eV) and only weakly dependent on the environment (cf Table 1). Overall, the *g* tensor reported for green UM in ref 45 cannot be reproduced within the KCZ model with physically reasonable parameter values. This is obviously related to the unusually high rhombicity of this *g* tensor, discussed above, as within the KCZ model the rhombicity is directly proportional to λ/*E*.

Raulin et al.^45^ compared their *g* values for S_2_^•–^ in UM (2.03, 1.85, 2.69) with the *g* values calculated for isolated S_2_^•–^ at the second-order DFT/BP86 level (2.034, 2.002, 2.858), taken from ref 84, and claimed that the two sets are similar. Based on this apparent similarity and also dissimilarity with the *g* values for S_2_^•–^ in alkali halides, they suggested that “the radical S_2_^–^ trapped inside sodalite cages is close to the free molecule, contrary to the S_2_^–^ in the sulfur-doped alkali halides.” There are two problems with this interpretation. First, as discussed above, the second-order DFT *g* values quoted above are *not* the correct *g* values for isolated S_2_^•–^, the correct ones are instead 0, 0, 4. Thus, based on the similarity of the *g* tensors, it is the S_2_^•–^ radical trapped in KCl or KBr that appears most similar to the free molecule (cf Table 3). Second, the *g* values reported for S_2_^•–^ in UM and those DFT-calculated for isolated S_2_^•–^ are actually not similar. Recall that the second-order method used to calculate the *g* tensor in ref 84, is unable to predict the *g*_*y*_ value smaller than *g*_*e*_, such as 1.85 reported in the experimental study. Consequently, the quoted DFT calculations also fail to explain the anomalously high rhombicity of δ = 0.18 of the experimental *g* tensor from ref 45, giving instead *δg* = 0.032. In fact, if the high rhombicity could be explained by the environment perturbing the trapped S_2_^•–^ radical, one had to assume that the perturbation should be an order of magnitude greater for S_2_^•–^ in UM than for S_2_^•–^ in NaCl, showing the highest rhombicity among the sulfur-doped alkali halides. This assumption seems unlikely upon looking at the geometry of the sodalite cage, where the shortest contact of S atoms with the Na atoms is 2.74 Å, compared with 2.55 Å for S_2_^•–^ trapped in NaCl lattice.

In conclusion of the above considerations, we believe that the assignment of the *g*_*x*,*y*_ tensor components for S_2_^•–^ in green UM reported in ref 45 is likely to be partially incorrect due to the very distinct values of the *g*_*x*_ and *g*_*y*_ components. Such high rhombicity of the *g* tensor strongly disagrees with the results of EPR experiments on S_2_^•–^ in alkali halides, our present ab initio calculations for extended cluster models, and the phenomenological KCZ model (using reasonable parameters).

The experimental spectrum of S_2_^•–^ in UM from ref 45 shows a broad signal, in which the presence of *g* values significantly below *g*_*e*_ (high-field) and significantly above *g*_*e*_ (low-field) is evidently recognized. This wide range of the *g* values is qualitatively corroborated by the present calculations although no quantitative agreement is found with any of the calculated cluster models. We note, however, that the *g* values reported in the experimental study represent the simplest explanation of the observed broad spectrum in terms of the single *g* tensor, and do not account for a possibility that the spectrum may result from the overlap of signals from several types of paramagnetic centers. For example, upon looking more carefully at Figure 6 in ref 45, one could notice the possibility of two broad peaks overlapping in the low-field region, suggesting that its explanation in terms of the single paramagnetic species (single *g* tensor) could be oversimplified. Such an intriguing possibility is strongly corroborated by the present calculations, showing a great variability of the *g* values for S_2_^•–^ with the change of its local environment. In particular, two notably different *g* tensors are predicted for the two considered sitings of S_2_^•–^ in sodalite cage of UM (models **1**L and **2**L). One could even notice that averaging the values calculated for the two models yields the lowest *g*_*y*_ and the highest *g*_*z*_ values in a reasonably good match with the corresponding experimental values (cf Table 3). Though such an interpretation is tempting, we cannot exclude that the good agreement is somewhat accidental, as only two final models of S_2_^•–^ bearing UM considered by us are likely not able to capture all the complexity of real UM samples, which may affect the sensitive EPR signal of S_2_^•–^ radical (different Al:Si ratios, substitutions of Al^3+^ and Na^+^ ions, lattice defects etc.). The middle *g*_*x*_ value reported by Raulin et al., 2.03(4), is also not far from the value of 1.991 calculated for one of the considered UM models (cf Table 3). However, this value of 2.03(4) is also, intriguingly, very close to both the middle component of S_3_^•–^ *g* tensor (2.034–2.038) and its *g*_iso_ (2.028–2.032) in resolved and unresolved EPR spectra, respectively.^16,45,46,84,96^ The S_3_^•–^ radical virtually always accompanies S_2_^•–^ one in green UMs, and the samples studied in ref 45 were no exception. Therefore, it may be difficult to fully separate a narrow signal of S_3_^•–^ from the broad signal of S_2_^•–^.

In a recent work on near-infrared emitting UM, Viola et al. claimed to observe S_2_^•–^ EPR spectra with a rhombic, but almost isotropic *g* tensor (2.002, 2.012, and 2.031),^97^ an assignment in a clear contradiction with the discussed computational and experimental data. We believe that the aforementioned signal must originate from some other S- or Cl-based radical: actually, the *g* tensor components reported in ref 97 are suspiciously close to the values reported earlier for S_2_O^•–^ radical (2.001, 2.011, 2.031).^96^ Another alleged EPR observation of S_2_^•–^ in sodalite lattice, this time more consistent with our results, comes from a recent study on thermally treated sapozhnikovite, i.e., sodalite group mineral hosting HS^–^ ions.^98^ Upon heating at 700 °C, the appearance of a broad band with *g*_iso_ > 2.1 was reported and ascribed to S_2_^•–^ radicals, as intermediates in the transformation of HS^–^ to S_3_^•–^. This assignment was subsequently supported by the second-order DFT calculations.^88^ The latter approach, although able to roughly estimate the anisotropy and trace of the *g* tensor for embedded S_2_^•–^, cannot properly reproduce *g* tensor components, as shown above.

Upon our calculations and critical analysis of available experimental data, we can conclude that the *g* tensor of S_2_^•–^ in UM (if resolved into components) should be anisotropic, almost axially symmetric (i.e., low rhombicity), with perpendicular component below 2.0, and the axial one greater than 2.3, resulting in the *g*_iso_ greater than 2.1. Therefore, the components and trace of the *g* tensor S_2_^•–^ are very different than the corresponding values for the accompanying S_3_^•–^ radical. Note that the small S_2_^•–^ radical is expected to rotate rather freely inside sodalite cages,^46^ thus, except at low temperatures, one can expect only the averaged signal to be detectable. Raulin et al. recorded the EPR spectra at 4 K, a temperature low enough to hinder the molecular rotation of S_2_^•–^. However, even such low-temperature conditions may not exclude the possibility of several slightly different sitings of S_2_^•–^ in the sodalite cage, exemplified by our cluster models **1** and **2**, that could coexist in the experiment. Due to the predicted sensitivity of S_2_^•–^ EPR signal to the local environment, we can expect that it may give somewhat different EPR signals in different UM samples, or even more than one signal within a single UM sample due to the coexistence of S_2_^•–^ radicals in different local environments, contributing to the broadness of the spectral lines (so-called *g*-strain).

Based on our studies, we thus propose that the lowest and the highest *g* values reported by Raulin et al. (1.85 and 2.69) indeed correspond to the nonaxial (*g*_*x*_ ≈ *g*_*y*_) and axial (*g*_*z*_) components, whereas the middle value (2.03) is most likely the *g*_iso_ of S_3_^•–^ radical, accompanying S_2_^•–^ in UMs. We suggest that the experimental EPR spectrum of S_2_^•–^ in green UM should be revisited, following the experimental protocol of Raulin et al. (i.e., liquid He temperature EPR measurements on Fe-free UM samples, with the presence of S2- confirmed additionally by UV–Vis and Raman spectroscopies), but considering the possibility of resolving this spectrum into a combination of several signals, to account for the strong environment-dependence of the *g* tensor, and the possibility of S_3_^•–^ admixture.

## 4 Conclusions

We performed DFT and CASSCF/CASPT2 calculations on S_2_^•–^ radical embedded in ultramarine lattices using large cluster models that incorporate the whole sodalite cage, with the aim of describing electronically excited states and the ground-state *g* tensor. The predicted wavelength of the most intense optical transition (∼ 390 nm), which is due to the π_*u*_ → π_g_^*^ electron excitation, was found to be in excellent agreement with the experimental UV–Vis spectra. At the CASPT2 level we also targeted other excited states of S_2_^•–^, some of which have significant multireference character, and analyzed changes in the excited state energies of the S_2_^•–^ radical caused by embedding. With regard to the most intense π_*u*_ → π_g_^*^ excitations, we observed a reasonable agreement between the CASPT2 and TDDFT results and only a small dependence on the details of the cluster model. The accurate prediction of the *g* tensor of S_2_^•–^ in UMs proved to be a more challenging task, with results strongly depending on both computational methods and the selected geometrical model. A common approach to calculate the *g* tensor via the linear response method based on spin-unrestricted DFT calculations struggles to properly capture strong spin–orbit coupling in systems with orbital near-degeneracy, and hence, such an approach is inappropriate for embedded S_2_^•–^ or similar diatomic radicals. (Obviously, it remains valid if the degeneracy of the partially filled π* orbitals is effectively lifted by covalent interactions, such as in the case of O_2_^•–^ ligand bonded to a transition metal ion.) Among the methods investigated in this study, only CASPT2 with a variational treatment of the spin–orbit coupling (herein referred to as the first-order approach) yields meaningful values of the *g* tensor for S_2_^•–^ radical, predicting the *g* tensor to be strongly anisotropic, but having only a small rhombicity, with the axial component and two nearly degenerated perpendicular components being, respectively, much larger (*g*_*z*_) and much smaller (*g*_*x*,*y*_) than the free electron value, *g*_*e*_. Such *g* values are consistent with the available experimental data for S_2_^•–^ in alkali metal halides and the phenomenological Känzig–Cohen–Zeller model for diatomic radicals. The specific values of the *g* tensor components are predicted to be very sensitive to the local environment of the radical, which possibly can lead to a certain scattering of the S_2_^•–^ EPR signals between various UM samples, or even the presence of several nonequivalent S_2_^•–^ signals in a single UM sample. Upon our results, the assignment of EPR signals with either a strongly isotropic *g* tensor,^97^ or with *g*_iso_ close to *g*_*e*_,^16^ should be considered incorrect. The most reliable measurements of S_2_^•–^ *g* tensor in ultramarines, up to our best knowledge, are those presented by Raulin et al.^45^ The high anisotropy of the experimental *g* tensor from ref 45 is corroborated by the present calculations (CASPT2, first-order approach), allowing to explain the presence of *g* values much larger than *g*_*e*_ and much smaller than *g*_*e*_, observed in the low- and high-field regions of the experimental spectrum, respectively. Nevertheless, the high rhombicity of the experimental *g* tensor reported in ref 45 is not reproduced in our calculations and, upon detailed analyses presented here, appears rather suspicious. Based on our results, we suggest the experimental report of the presumed S_2_^•–^ middle component (∼2.0) should be likely reassigned to S_3_^•–^, whereas the low- and high-field regions of the spectrum could contain overlapped signals from S_2_^•–^ radical siting in slightly different local environments. Regarding future experimental studies, we recommend that the EPR identification of S_2_^•–^ in solids should be supported by other spectroscopic evidence, e.g., the well-established visible absorption (about 400 nm) and Raman signatures for this diatomic radical anion (about 590 cm^–1^).

## Supplementary Material





## Notes

Geometries of the cluster models in the .xyz format (TXT)
Additional figure, tables, and further computational details (PDF)
