# Supplementary material for: Optical and Magnetic Properties of Yellow Chromophore S2 •– in Ultramarine Pigments: CASPT2 and DFT Studies
Source: J Phys Chem A. 2026 Aug 14;130(34):6744–58. doi: 10.1021/acs.jpca.6c03753 (PMC13528282; doi:10.1021/acs.jpca.6c03753)
Supplement: Supplementary file 1 [file jp6c03753_si_002.pdf]

Supporting Information: Optical and Magnetic Properties of Yellow Chromophore $S_2^{\bullet-}$ in Ultramarine Pigments: CASPT2 and DFT studies

Paweł Rejmak,*,† Mariusz Radoń,*,‡ and Piotr Pietrzyk‡

†*Institute of Physics Polish Academy of Sciences, Al. Lotników 32/46, PL-02668 Warsaw, Poland*

‡*Jagiellonian University, Faculty of Chemistry, Gronostajowa 2, PL-30-387 Kraków, Poland*

E-mail: rejmakp@gmail.com,rejmak@ifpan.edu.pl; mradon@chemia.uj.edu.pl

List of Tables

S1 Considered electronic states of $S_2^{\bullet -}$ . . . S4

S2 Detailed CASSCF/CASPT2 results for isolated $S_2^{\bullet -}$ . . . S8

S3 Detailed CASSCF/CASPT2 results for ($S_2^{\bullet -}$)Na_7-*sod* (large) . . . S9

S4 Detailed CASSCF/CASPT2 results for ($S_2^{\bullet -}$)Na_8Cl-*sod* (large) . . . S10

S5 Definition of basis sets used in this study. . . . S12

S6 Basis-set dependence of CASPT2 energies, ($S_2^{\bullet -}$)Na_7-*sod* (medium) . . . S12

S7 Basis-set dependence of CASPT2 energies, ($S_2^{\bullet -}$)Na_8Cl-*sod* (medium) . . . S12

S8 Basis-set dependence of CASPT2 energies, isolated $S_2^{\bullet -}$ . . . S13

S9 CASPT2 energies of higher excited states . . . S14

S10 CASPT2 and MS-CASPT2 excitation energies . . . S15

S11 Vertical excitation energies for isolated $S_2^{\bullet - a,b}$ . . . S15

S12 Calculated S–S bond lengths (Å) . . . S16

S13 Energy Difference Between $^2\Pi_g^{(1),(2)}$ States or $\pi^*_{x,y}$ Orbitals (eV). . . . S17

S14 Comparison of BP86 g values with various basis sets . . . S18

S15 Comparison of TDDFT excitation energies with and without TDA . . . S18

List of Figures

S1 $^2\Pi_g \rightarrow {}^2\Pi_u$ vertical excitation energy of $S_2^{\bullet -}$ as a function of the S–S distance. S16

S1 Electronic states of ${S_2}^{\bullet -}$

Table S1 lists principal configuration state functions (CSFs) for the considered electronic states of ${S_2}^{\bullet -}$: the ground state $^2\Pi_g$ (D_0, D_1); the $\pi_u \to \pi_g^*$ excited state $^2\Pi_u$ (D_2, D_3), the $\pi_g \to \sigma_u^*$ excited states: $^2\Delta_u$ (D_4, D_5), $^2\Sigma_u^-$ (D_6), $^2\Sigma_u^+$ (D_7) and $^4\Sigma_u^-$ (Q_1); the $\sigma_g \to \pi_g^*$ excited state $^2\Sigma_g^+$ (D_8).

We continue using the term symbols of isolated ${S_2}^{\bullet -}$ (e.g., $^2\Pi_g$, $^2\Sigma_u^+$) even in less symmetric cluster models to indicate the principal electronic parentage of a given state. Note that symbols D_0 to D_8 always refer to the electronic parentage of a given doublet state, not to their energy ordering (which is model-dependent).

The CSFs are specified by occupations of the active orbitals: 2 for doubly occupied, 0 for unoccupied, and ↑≡u / ↓≡d for singly occupied. (Only the occupations are specified in the short notation assuming the order of active orbitals as in Table S1.) When reading the CSF symbol from left to right, the spin coupling with any singly occupied orbital produces an eigenfunction of the $\hat{S}^2$ operator with the total spin quantum number either increased (↑) or decreased (↓) by 1/2. The obtained CSFs may be combinations of several Slater determinants, e.g.,

$$|(\pi_x^*)^{\uparrow}(\pi_y^*)^{\downarrow}(\sigma^*)^{\uparrow}\rangle = \tfrac{1}{\sqrt{2}}\left|\overset{+}{\pi_x^*}\overset{-}{\pi_y^*}\overset{+}{\sigma^*}\right| - \tfrac{1}{\sqrt{2}}\left|\overset{-}{\pi_x^*}\overset{+}{\pi_y^*}\overset{+}{\sigma^*}\right| \tag{S.1}$$

$$|(\pi_x^*)^{\uparrow}(\pi_y^*)^{\uparrow}(\sigma^*)^{\downarrow}\rangle = \sqrt{\tfrac{2}{3}}\left|\overset{+}{\pi_x^*}\overset{+}{\pi_y^*}\overset{-}{\sigma^*}\right| - \tfrac{1}{\sqrt{6}}\left|\overset{+}{\pi_x^*}\overset{-}{\pi_y^*}\overset{+}{\sigma^*}\right| - \tfrac{1}{\sqrt{6}}\left|\overset{-}{\pi_x^*}\overset{+}{\pi_y^*}\overset{+}{\sigma^*}\right| \tag{S.2}$$

Table S1: Considered electronic states of $S_2^{\bullet -}$.

term	state	principal configuration (long notation)	(short notation)
$^2\Pi_g$	$D_0 \equiv {}^2\Pi_g^{(1)}$	$\left\|(\sigma)^2(\pi_x)^2(\pi_y)^2(\pi_x^*)^2(\pi_y^*)^{\uparrow}(\sigma^*)^0\right\rangle$	2222u0
	$D_1 \equiv {}^2\Pi_g^{(2)}$	$\left\|(\sigma)^2(\pi_x)^2(\pi_y)^2(\pi_x^*)^{\uparrow}(\pi_y^*)^2(\sigma^*)^0\right\rangle$	222u20
$^2\Pi_u$	$D_2 \equiv {}^2\Pi_u^{(1)}$	$\left\|(\sigma)^2(\pi_x)^2(\pi_y)^{\uparrow}(\pi_x^*)^2(\pi_y^*)^2(\sigma^*)^0\right\rangle$	22u220
	$D_3 \equiv {}^2\Pi_u^{(2)}$	$\left\|(\sigma)^2(\pi_x)^{\uparrow}(\pi_y)^2(\pi_x^*)^2(\pi_y^*)^2(\sigma^*)^0\right\rangle$	2u2220
$^2\Delta_u$	$D_4 \equiv {}^2\Delta_u^{(1)}$	$\frac{1}{\sqrt{2}}\cdot\left\|(\sigma)^2(\pi_x)^2(\pi_y)^2(\pi_x^*)^2(\pi_y^*)^0(\sigma^*)^{\uparrow}\right\rangle$ $-\frac{1}{\sqrt{2}}\cdot\left\|(\sigma)^2(\pi_x)^2(\pi_y)^2(\pi_x^*)^0(\pi_y^*)^2(\sigma^*)^{\uparrow}\right\rangle$	22220u − 22202u
	$D_5 \equiv {}^2\Delta_u^{(2)}$	$\left\|(\sigma)^2(\pi_x)^2(\pi_y)^2(\pi_x^*)^{\uparrow}(\pi_y^*)^{\downarrow}(\sigma^*)^{\uparrow}\right\rangle$	222udu
$^2\Sigma_u^-$	D_6	$\left\|(\sigma)^2(\pi_x)^2(\pi_y)^2(\pi_x^*)^{\uparrow}(\pi_y^*)^{\uparrow}(\sigma^*)^{\downarrow}\right\rangle$	222uud
$^2\Sigma_u^+$	D_7	$\frac{1}{\sqrt{2}}\cdot\left\|(\sigma)^2(\pi_x)^2(\pi_y)^2(\pi_x^*)^2(\pi_y^*)^0(\sigma^*)^{\uparrow}\right\rangle$ $+\frac{1}{\sqrt{2}}\cdot\left\|(\sigma)^2(\pi_x)^2(\pi_y)^2(\pi_x^*)^0(\pi_y^*)^2(\sigma^*)^{\uparrow}\right\rangle$	22220u + 22202u
$^2\Sigma_g^+$	D_8	$\left\|(\sigma)^u(\pi_x)^2(\pi_y)^2(\pi_x^*)^2(\pi_y^*)^2(\sigma^*)^0\right\rangle$	u22220
$^4\Sigma_u^-$	Q_1	$\left\|(\sigma)^2(\pi_x)^2(\pi_y)^2(\pi_x^*)^{\uparrow}(\pi_y^*)^{\uparrow}(\sigma^*)^{\uparrow}\right\rangle$	222uuu

Deriving the principal configurations for the $^2\Pi_g$, $^2\Pi_u$, $^2\Sigma_g^+$, and $^4\Sigma_u^-$ states of $S_2^{\bullet-}$ is straightforward. For the remaining states originating from the $(\pi^*)^2(\sigma^*)^1$ configuration, it is helpful to consider complex-valued $\pi^*_{\pm1}$ orbitals being combinations of the real-valued $\pi^*_{x,y}$ ones

$$\pi^*_{\pm1} = \frac{1}{\sqrt{2}} \left\{ \pi^*_x \pm i\pi^*_y \right\}. \tag{S.3}$$

The complex-valied orbitals are eigenfunctions of $\hat{\Lambda}$, the total angular momentum along the molecular z-axis with the eigenvalues ±1, i.e.,

$$\hat{\Lambda}\,\pi_{\pm1} = \pm\pi_{\pm1}. \tag{S.4}$$

The σ^* orbital is also an eigenfunction of the same operator, but corresponding to the zero eigenvalue, i.e.

$$\hat{\Lambda}\,\sigma = 0. \tag{S.5}$$

The $^2\Delta_u$ term has two components being eigenstates of $\hat{\Lambda}$ with eigenvalues $\lambda = \pm2$, i.e.,

$$\hat{\Lambda}\ \left|^2\Delta_u,\, \lambda = \pm2\right\rangle = \pm2 \cdot \left|^2\Delta_u,\, \lambda = \pm2\right\rangle. \tag{S.6}$$

Thus, based on (S.4), (S.5), and (S.6), the two components of the $^2\Delta_u$ state must originate from either π_1 or π_{-1} being doubly occupied, and the third unpaired electron in σ^*. The two components of the degenerate $^2\Delta_u$ state must be then:

$$\left|^2\Delta_u,\, \lambda = +2\right\rangle = \left| \overset{+}{\pi}{}^*_{+1} \overset{-}{\pi}{}^*_{+1} \overset{+}{\sigma}{}^* \right| \tag{S.7}$$

$$\left|^2\Delta_u,\, \lambda = -2\right\rangle = \left| \overset{+}{\pi}{}^*_{-1} \overset{-}{\pi}{}^*_{-1} \overset{+}{\sigma}{}^* \right| \tag{S.8}$$

$$\tag{S.9}$$

Expressing these wave functions in terms of the real-valued $\pi^*_{x,y}$ orbitals, using eq. (S.3),

leads to the following expressions:

$$|^2\Delta_u,\,\lambda=\pm 2\rangle = \tfrac{1}{2}\left|\overset{+}{\pi_x^*}\overset{-}{\pi_x^*}\overset{+}{\sigma^*}\right| \pm \tfrac{i}{2}\left|\overset{+}{\pi_x^*}\overset{-}{\pi_y^*}\overset{+}{\sigma^*}\right| \mp \tfrac{i}{2}\left|\overset{-}{\pi_x^*}\overset{+}{\pi_y^*}\overset{+}{\sigma^*}\right| - \tfrac{1}{2}\left|\overset{+}{\pi_y^*}\overset{-}{\pi_y^*}\overset{+}{\sigma^*}\right| \tag{S.10}$$

The normalized real part is thus

$$^2\Delta_u^{(1)} = \tfrac{1}{\sqrt{2}}\left\{\left|\overset{+}{\pi_x^*}\overset{-}{\pi_x^*}\overset{+}{\sigma^*}\right| - \left|\overset{+}{\pi_y^*}\overset{-}{\pi_y^*}\overset{+}{\sigma^*}\right|\right\} \tag{S.11}$$

and the normalized imaginary part is

$$^2\Delta_u^{(2)} = \tfrac{1}{\sqrt{2}}\left\{\left|\overset{+}{\pi_x^*}\overset{-}{\pi_y^*}\overset{+}{\sigma^*}\right| - \left|\overset{-}{\pi_x^*}\overset{+}{\pi_y^*}\overset{+}{\sigma^*}\right|\right\} = \left|(\pi_x^*)^{\uparrow}(\pi_y^*)^{\downarrow}\,(\sigma^*)^{\uparrow}\right| \tag{S.12}$$

These are identical as given above in Table S1.

Analogously, the $^2\Sigma_u^+$ term must satisfy

$$\hat{\Lambda}\,|^2\Sigma_u^+\rangle = 0. \tag{S.13}$$

Knowing that it is derived from the $(\pi^*)^2(\sigma^*)^1$ configuration, therefore, leads to conclusion that both π_{+1}^* and π_{-1}^* complex orbitals must be singly occupied, coupled antiferromagnetically, with the third unpaired electron must occupy σ^*, i.e.

$$|^2\Sigma_u^+\rangle = \left|(\pi_{+1}^*)^{\uparrow}(\pi_{-1}^*)^{\downarrow}\,(\sigma^*)^{\uparrow}\right| = \tfrac{1}{\sqrt{2}}\left\{\left|\overset{+}{\pi_{+1}^*}\overset{-}{\pi_{-1}^*}\overset{+}{\sigma^*}\right| - \left|\overset{-}{\pi_{+1}^*}\overset{+}{\pi_{-1}^*}\overset{+}{\sigma^*}\right|\right\}. \tag{S.14}$$

Expressing this wave function in terms of the real-valued $\pi_{x,y}^*$ orbitals, using (S.3), leads to

the following expression

$$
\begin{aligned}
|{}^2\Sigma_u^+\rangle &= \frac{1}{\sqrt{2}}\left\{\frac{1}{2}\left|\overset{+}{\pi_x^*}\overset{-}{\pi_x^*}\overset{+}{\sigma^*}\right| - \frac{i}{2}\left|\overset{+}{\pi_x^*}\overset{-}{\pi_y^*}\overset{+}{\sigma^*}\right| + \frac{i}{2}\left|\overset{+}{\pi_y^*}\overset{-}{\pi_x^*}\overset{+}{\sigma^*}\right| + \frac{1}{2}\left|\overset{+}{\pi_y^*}\overset{-}{\pi_y^*}\overset{+}{\sigma^*}\right|\right\} \\
&- \frac{1}{\sqrt{2}}\left\{\frac{1}{2}\left|\overset{-}{\pi_x^*}\overset{+}{\pi_x^*}\overset{+}{\sigma^*}\right| - \frac{i}{2}\left|\overset{-}{\pi_x^*}\overset{+}{\pi_y^*}\overset{+}{\sigma^*}\right| + \frac{i}{2}\left|\overset{-}{\pi_y^*}\overset{+}{\pi_x^*}\overset{+}{\sigma^*}\right| + \frac{1}{2}\left|\overset{-}{\pi_y^*}\overset{+}{\pi_y^*}\overset{+}{\sigma^*}\right|\right\} \\
&= \frac{1}{\sqrt{2}}\left\{\left|\overset{+}{\pi_x^*}\overset{-}{\pi_x^*}\overset{+}{\sigma^*}\right| + \left|\overset{+}{\pi_y^*}\overset{-}{\pi_y^*}\overset{+}{\sigma^*}\right|\right\}, \qquad \text{(S.15)}
\end{aligned}
$$

which is identical with the wave function given above in Table S1.

S2 Details of CASSCF/CASPT2 Calculations

S2.1 Energies and Wave Functions

Tables S2–S4 report the energies and CAS CI expansions for isolated $S_2^{\bullet-}$ as well as $(S_2^{\bullet-})Na_7$-*sod* (large, **1**L) and $(S_2^{\bullet-})Na_8Cl$-*sod* (large, **2**L) cluster models.

Table S2: Detailed CASSCF/CASPT2 results for isolated $S_2^{\bullet -}$: total energies and composition of the CASSCF wave function. [a]

term	state	E_{CASSCF} [b]	E_{CASPT2} [b]	CSF [c]	coefficient [d]
$^2\Pi_g$	D_0	−797.294 595	−797.644 833	2222u0	−0.987 58
				u2d22u	−0.077 72
				0222u2	−0.053 81
				u2u22d	0.120 26
	D_1	−797.294 595	−797.644 834	222u20	0.987 58
				ud222u	0.077 72
				022u22	0.053 81
				uu222d	−0.120 26
$^2\Delta_u$	D_4	−797.184 541	−797.538 795	22220u	0.674 51
				22202u	−0.674 51
				22022u	−0.203 32
				20222u	0.203 32
	D_5	−797.184 541	−797.538 253	222udu	−0.953 91
				2ud22u	0.287 54
$^2\Sigma_u^-$	D_6	−797.181 062	−797.545 465	222uud	0.968 96
				u2du22	−0.065 59
				ud22u2	0.065 59
				2uu22d	−0.226 03
$^2\Pi_u$	D_2	−797.175 288	−797.527 933	22u220	−0.955 72
				u222du	−0.179 43
				u222ud	0.225 24
	D_3	−797.175 288	−797.527 933	2u2220	0.955 72
				u22d2u	0.179 43
				u22u2d	−0.225 24
$^2\Sigma_u^+$	D_7	−797.170 072	−797.523 327	22220u	−0.663 89
				22202u	−0.663 89
				22022u	0.235 44
				20222u	0.235 44
$^2\Sigma_g^+$	D_8	−797.143 387	−797.486 821	22u2ud	0.109 64
				2u2u2d	0.109 64
				u22220	0.985 35
$^4\Sigma_u^-$	Q_1	−797.217 833	−797.560 548	222uuu	0.967 72
				2uu22u	0.240 72
				u2u2u2	−0.052 75
				uu2u22	0.052 75

[a] Using basis set aT/D (see Table S5). [b] Value in hartree. [c] CSF in terms of active orbitals (short notation). [d] Coefficient multiplying a given CSF in the CASSCF wave function, only reported if > 0.05 in magnitude.

Table S3: Detailed CASSCF/CASPT2 results for ($S_2^{\bullet -}$)Na_7-*sod* (large): total energies and composition of the CASSCF wave function. [a]

term	state	E_{CASSCF} [b]	E_{CASPT2} [b]	CSF [c]	coefficient [d]
$^2\Pi_g$	D_0	−12 828.756 251	−12 842.058 252	2222u0	−0.983 01
				u2d22u	0.085 39
				0222u2	−0.089 78
				u2u22d	−0.122 84
	D_1	−12 828.746 864	−12 842.049 716	222u20	−0.982 43
				ud222u	0.087 45
				022u22	−0.089 54
				uu222d	−0.125 22
$^2\Pi_u$	D_2	−12 828.629 048	−12 841.937 209	22u220	0.959 19
				u222du	−0.179 56
				u222ud	0.191 95
				02u222	0.069 14
	D_3	−12 828.623 900	−12 841.932 226	2u2220	−0.959 42
				u22d2u	0.178 95
				u22u2d	−0.193 36
				0u2222	−0.069 81
$^2\Sigma_g^+$	D_8	−12 828.593 914	−12 841.905 066	u22220	−0.960 39
				22220u	−0.230 04
				22022u	0.068 39
				22202u	0.066 64
				22u2ud	0.063 74
				2u2u2d	0.054 57
$^2\Delta_u$	D_4	−12 828.590 417	−12 841.908 578	22220u	−0.853 47
				22202u	0.343 93
				u22220	0.244 60
				22022u	0.252 71
				22u2du	−0.093 46
				20222u	−0.084 73
				u2u2d2	−0.076 07
				u2d2u2	−0.062 75
	D_5	−12 828.587 714	−12 841.906 607	222udu	0.954 06
				2ud22u	−0.266 62
				2u22du	0.070 21
				ud22u2	0.052 05
				u2ud22	0.061 62
				uu22d2	0.062 67
$^2\Sigma_u^-$	D_6	−12 828.573 674	−12 841.901 550	222uud	−0.969 64
				u2du22	−0.088 35
				ud22u2	0.090 02
				2uu22d	0.197 77
$^2\Sigma_u^+$	D_7	−12 828.567 690	−12 841.886 533	22202u	−0.879 76
				22220u	−0.334 51
				22022u	0.128 68
				20222u	0.288 01
				ud2u22	−0.063 66

Continued on next page

Table S3 (continued).

	state	E_{CASSCF} [b]	E_{CASPT2} [b]	CSF [c]	coefficient [d]
				uu2d22	−0.073 10
$^4\Sigma_u^-$	Q_1	−12 828.626 507	−12 841.935 070	222uuu	−0.971 51
				2uu22u	0.200 50
				u2uu22	−0.086 31
				uu22u2	0.091 37

[a] Using basis set aT/D (see Table S5). [b] Value in hartree. [c] CSF in terms of active orbitals (short notation). [d] Coefficient multiplying a given CSF in the CASSCF wave function, only reported if > 0.05 in magnitude.

Table S4: Detailed CASSCF/CASPT2 results for ($S_2^{\bullet-}$)Na_8Cl-*sod* (large): total energies and composition of the CASSCF wave function. [a]

term	state	E_{CASSCF} [b]	E_{CASPT2} [b]	CSF [c]	coefficient [d]
$^2\Pi_g$	D_0	−12 991.252 796	−13 004.596 604	2222u0	−0.982 09
				uu222d	−0.098 97
				0222u2	−0.087 12
				ud222u	0.069 94
				u2u22d	−0.068 35
	D_1	−12 991.249 164	−13 004.593 552	222u20	0.981 80
				u2u22d	0.105 22
				022u22	0.086 94
				u2d22u	−0.063 72
				uu222d	−0.068 02
$^2\Pi_u$	D_2	−12 991.126 618	−13 004.476 467	22u220	−0.957 99
				u22u2d	−0.149 90
				u22d2u	0.137 06
				u222ud	−0.107 03
				u222du	0.100 12
				02u222	−0.068 27
				2u2220	−0.065 58
	D_3	−12 991.122 420	−13 004.472 622	2u2220	0.956 07
				u222ud	0.151 91
				u222du	−0.146 10
				u22u2d	−0.106 07
				u22d2u	0.099 86
				0u2222	0.067 75
				22u220	−0.067 04
$^2\Sigma_g^+$	D_8	−12 991.091 795	−13 004.443 439	u22220	0.988 35
				22uu2d	−0.063 82
				2u22ud	−0.053 32
$^2\Delta_u$	D_4	−12 991.089 454	−13 004.449 523	22220u	−0.786 07
				22202u	0.528 45
				2ud22u	0.240 78
				222udu	−0.134 44

Continued on next page

Table S4 (continued).

state		E_{CASSCF} [b]	E_{CASPT2} [b]	CSF [c]	coefficient [d]
				20222u	0.102 07
				uu22d2	−0.055 04
	D_5	−12 991.088 846	−13 004.449 621	222udu	0.946 46
				22022u	−0.176 22
				20222u	0.170 40
				22202u	0.108 28
				22220u	−0.090 16
				2ud22u	−0.084 95
$^2\Sigma_u^-$	D_6	−12 991.075 011	−13 004.444 286	222uud	−0.970 17
				u2d2u2	0.066 47
				ud2u22	−0.074 75
				2uu22d	−0.190 04
$^2\Sigma_u^+$	D_7	−12 991.072 233	−13 004.432 866	22220u	−0.526 87
				22202u	−0.779 97
				22022u	0.224 70
				u2d22u	−0.053 67
				20222u	0.204 45
				02222u	0.056 15
				u2ud22	−0.054 22
$^4\Sigma_u^-$	Q_1	−12 991.128 351	−13 004.477 757	222uuu	0.971 20
				2uu22u	0.201 58
				u2uu22	−0.088 57
				uu22u2	−0.091 03

[a] Using basis set aT/D (see Table S5). [b] Value in hartree. [c] CSF in terms of active orbitals (short notation). [d] Coefficient multiplying a given CSF in the CASSCF wave function, only reported if > 0.05 in magnitude.

S2.2 Choice of basis set in CASPT2 calculations

Table S5 lists the tested basis sets, of which the default basis set used in the main article is aT/D. Only valence electron were correlated for O, S, Al, Si; i.e., all 1s orbitals of O as well as 1s2s2p orbitals of S, Al, Si were put in the frozen core (FC). Concerning Na, it was observed that in larger cluster models (1b), the Na 2p outer-core orbitals appear at similar energies as valence orbitals, but Na 2s are at least 1 a.u. lower in energy. Therefore, two choices of the FC were considered for Na atoms: 1s or 1s2s (cf Table S5).

The effect of basis set on the excitation energies was tested for medium-size cluster models of $(S_2^{\bullet-})Na_7$-*sod* (Table S6) and $(S_2^{\bullet-})Na_8Cl$-*sod* (Table S7) as well as for isolated $S_2^{\bullet-}$ (Table S8).

Table S5: Definition of basis sets used in this study.

basis	S	O,H,Si,Al	Na	FC (Na) [a]
T	cc-pV(T+d)Z-DK	cc-pVTZ-DK	cc-pwCVTZ-DK	1s
T/D″	cc-pV(T+d)Z-DK	cc-pVDZ-DK	cc-pwCVDZ-DK	1s [b]
T/D′	cc-pV(T+d)Z-DK	cc-pVDZ-DK	cc-pwCVDZ-DK	1s2s [b]
T/D	cc-pV(T+d)Z-DK	cc-pVDZ-DK	cc-pVDZ-DK	1s2s
aT/D	aug-cc-pV(T+d)Z-DK	cc-pVDZ-DK	cc-pVDZ-DK	1s2s
aQ/D	aug-cc-pV(Q+d)Z-DK	cc-pVDZ-DK	cc-pVDZ-DK	1s2s

[a] Frozen core of Na atoms. [b] Note that T/D′ and T/D″ only differ in the Na frozen-core.

Table S6: Basis-set dependence of CASPT2 energies for ($S_2{}^{\bullet-}$)Na_7-*sod* (medium) cluster. [a]

state		T	T/D″	T/D′	T/D	aT/D	aQ/D
$^2\Pi_g$	D_0	0.00	0.00	0.00	0.00	0.00	0.00
	D_1	0.21	0.21	0.21	0.21	0.20	0.20
$^2\Pi_u$	D_2	3.27	3.28	3.28	3.29	3.28	3.27
	D_3	3.40	3.41	3.41	3.42	3.41	3.40
$^2\Delta_u$	D_4	4.12	4.14	4.14	4.15	4.13	4.12
	D_5	4.16	4.19	4.19	4.20	4.17	4.16
$^2\Sigma_u^-$	D_6	4.31	4.33	4.33	4.34	4.31	4.29
$^2\Sigma_u^+$	D_7	4.69	4.71	4.71	4.72	4.69	4.68
$^2\Sigma_g^+$	D_8	4.16	4.17	4.17	4.18	4.17	4.16
$^4\Sigma_u^-$	Q_1	3.39	3.40	3.40	3.40	3.39	3.41

[a] All values in eV.

Table S7: Basis-set dependence of CASPT2 energies for ($S_2{}^{\bullet-}$)Na_8Cl-*sod* (medium) cluster. [a]

state		T	T/D″	T/D′	T/D	aT/D	aQ/D
$^2\Pi_g$	D_0	0.00	0.00	0.00	0.00	0.00	0.00
	D_1	0.10	0.09	0.09	0.09	0.09	0.09
$^2\Pi_u$	D_2	3.29	3.29	3.29	3.29	3.29	3.29
	D_3	3.38	3.39	3.39	3.39	3.39	3.38
$^2\Delta_u$	D_4	4.02	4.04	4.05	4.05	4.03	4.02
	D_5	4.02	4.04	4.04	4.05	4.03	4.02
$^2\Sigma_u^-$	D_6	4.16	4.19	4.19	4.20	4.17	4.16
$^2\Sigma_u^+$	D_7	4.48	4.50	4.50	4.51	4.48	4.47
$^2\Sigma_g^+$	D_8	4.18	4.19	4.19	4.19	4.19	4.18
$^4\Sigma_u^-$	Q_1	3.24	3.26	3.26	3.26	3.25	3.27

[a] All values in eV.

Table S8: Basis-set dependence of CASPT2 energies for isolated $S_2^{\bullet-}$. [a]

state		T	aT	aQ
$^2\Pi_g$	D_0	0.00	0.00	0.00
	D_1	0.00	0.00	0.00
$^2\Pi_u$	D_2	3.20	3.18	3.19
	D_3	3.20	3.18	3.19
$^2\Delta_u$	D_4	3.56	2.90	2.88
	D_5	3.58	2.89	2.87
$^2\Sigma_u^-$	D_6	3.70	2.70	2.64
$^2\Sigma_u^+$	D_7	3.98	3.31	3.29
$^2\Sigma_g^+$	D_8	4.27	4.30	4.31
$^4\Sigma_u^-$	Q_1	2.78	2.29	2.38

For the two cluster models (Tables S6 and S7), the following observations can be made:

- The excitation energies obtained using basis set T and T/D″, are identical to within 0.03 eV, showing that reduction from full TZ-quality on all atoms to DZ-quality on the atoms outside the inner $S_2^{\bullet-}$ is a justified approximation.

- Whether the FC for Na is 1s (T/D″) or 1s2s (T/D′) makes a very little difference. The same holds true about the effect of either cc-pVDZ-DK (T/D) or cc-pwCVDZ-DK (T/D′) for Na atoms.

- The effect of adding diffuse functions on S atoms, i.e. the difference between the T/D and aT/D results, is within 0.03 eV for the cluster models. The effect of subsequent increasing the basis set from TZ- to QZ-quality on S atoms, i.e., the difference between the aT/D and aQ/D results, is within 0.02 eV. It should be noticed, however, that for isolated $S_2^{\bullet-}$ the effect of diffuse functions is definitely more important (cf Table S8).

Further calculations are therefore performed using the aT/D basis set to enable comparison of selected results between the cluster models and isolated $S_2^{\bullet-}$, although the benefits from adding diffuse functions are rather limited for the cluster models and, therefore, if only these models were considered, even the smaller basis set T/D would be sufficient.

S2.3 Higher excited states

In our calculations we normally considered only 9 lowest doublets ($^2\Pi_g$, $^2\Pi_u$, $^2\Delta_u$, $^2\Sigma_u^-$, $^2\Sigma_u^+$, $^2\Sigma_g^+$) and 1 quartet state ($^4\Sigma_u^-$). Test calculations with 20 doublets and 4 quartets were performed for ($S_2^{\bullet -}$)Na_7-*sod*, ($S_2^{\bullet -}Na_8Cl$-*sod* (medium-size clusters), and for isolated $S_2^{\bullet -}$ (Table S9). It can be observed that higher doublet states (D_9 onward) and higher quartet states (Q_2 onward) have energies greater than 5.9 eV in both considered cluster models and greater than 4.5 eV in isolated $S_2^{\bullet -}$. The gap between the lowest excluded state (Q_2 or D_9) and the highest considered state (D_8) is always greater than 1.2 eV.

Table S9: CASPT2 energies of higher excited states. [a,b]

state		($S_2^{\bullet -}$)Na_7-*sod* (medium)	($S_2^{\bullet -}$)Na_8C-*sod* (medium)	$S_2^{\bullet -}$
$^2\Pi_g$	D_0	0.00	0.00	0.00
	D_1	0.20	0.09	0.00
$^2\Pi_u$	D_2	3.28	3.28	3.19
	D_3	3.40	3.38	3.19
$^2\Delta_u$	D_4	4.11	4.00	2.87
	D_5	4.15	4.01	2.85
$^2\Sigma_u^-$	D_6	4.29	4.16	2.64
$^2\Sigma_u^+$	D_7	4.67	4.46	3.27
$^2\Sigma_g^+$	D_8	4.14	4.20	4.33
[c]	D_9	6.18	6.11	4.90
[c]	D_{10}	6.89	6.94	5.39
[c]	D_{11}	7.03	6.98	5.42
[c]	D_{12}	7.23	7.10	5.51
[c]	D_{13}	7.91	7.79	6.37
[c]	D_{14}	8.11	7.89	6.37
[c]	D_{15}	8.12	8.44	7.12
[c]	D_{16}	8.58	7.94	7.12
[c]	D_{17}	8.76	8.51	6.24
[c]	D_{18}	8.85	8.74	7.29
[c]	D_{19}	8.82	8.73	7.33
$^4\Sigma_u^-$	Q_1	3.41	3.27	2.38
[c]	Q_2	5.94	5.99	5.10
[c]	Q_3	6.06	5.98	5.13
[c]	Q_4	6.29	6.13	5.23

[a]Values in eV. [b]Basis set aT/D. [c]Term symbol was not identified for higher states.

S2.4 Comparison between CASPT2 and MS-CASPT2 results

Table S10: CASPT2 and MS-CASPT2 excitation energies [a,b].

state		$(S_2^{\bullet-})Na_7$-*sod* (medium)		$S_2^{\bullet-}$	
		CASPT2	MS-CASPT2	CASPT2	MS-CASPT2
$^2\Pi_g$	D_0	0.00	0.00	0.00	0.00
	D_1	0.20	0.20	0.00	0.00
$^2\Pi_u$	D_2	3.28	3.28	3.18	3.18
	D_3	3.41	3.41	3.18	3.18
$^2\Delta_u$	D_4	4.13	4.13	2.90	2.90
	D_5	4.17	4.17	2.89	2.89
$^2\Sigma_u^-$	D_6	4.31	4.31	2.70	2.70
$^2\Sigma_u^+$	D_7	4.69	4.69	3.31	3.31
$^2\Sigma_g^+$	D_8	4.17	4.17	4.30	4.30

[a]Values in eV. [b]Basis set aT/D.

It was checked for one of the cluster models, $(S_2^{\bullet-})Na_7$-*sod* (medium-size), and isolated $S_2^{\bullet-}$, in both cases using basis set aT/D, that CASPT2 and MS-CASPT2 excitation energies are identical to within 0.01 eV (Table S10). Therefore, only ordinary CASPT2 energies (i.e., no multistate corrections) are considered in this study.

S2.5 Comparison with Other Correlated Methods for $S_2^{\bullet-}$

Table S11: Vertical excitation energies for isolated $S_2^{\bullet-}$ [a,b]

state	CASPT2[c] ($\epsilon = 0.25$)	CASPT2[c] ($\epsilon = 0$)	NEVPT2[d]	MRCI+Q[e]	CCSD(T)[f]
$^2\Pi_u$	3.18	3.12	3.21	3.10	3.09
$^2\Delta_u$	2.90	2.55	3.02	3.01	[g]
$^2\Sigma_u^-$	2.70	2.27	2.75	2.79	[g]
$^2\Sigma_u^+$	3.31	2.98	3.51	3.40	[g]
$^2\Sigma_g^+$	4.30	4.27	4.33	4.01	4.01

[a]Values in eV with respect to the $^2\Pi_g$ ground state. [b]Single-point calculations for PBE/ma-def2-TZVP geometry (S–S distance 2.020 Å) using aug-cc-pV(T+d)Z-DK basis set. [c]In parenthesis: IPEA shift parameter (ϵ), value in a.u. [d]Partially contracted. [e]With Davidson–Silver–Siegbahn size-consistency correction. [f]ROHF/UCCSD(T). [g]Calculations not performed as the state of interest is not properly represented by an ROHF-type determinant.

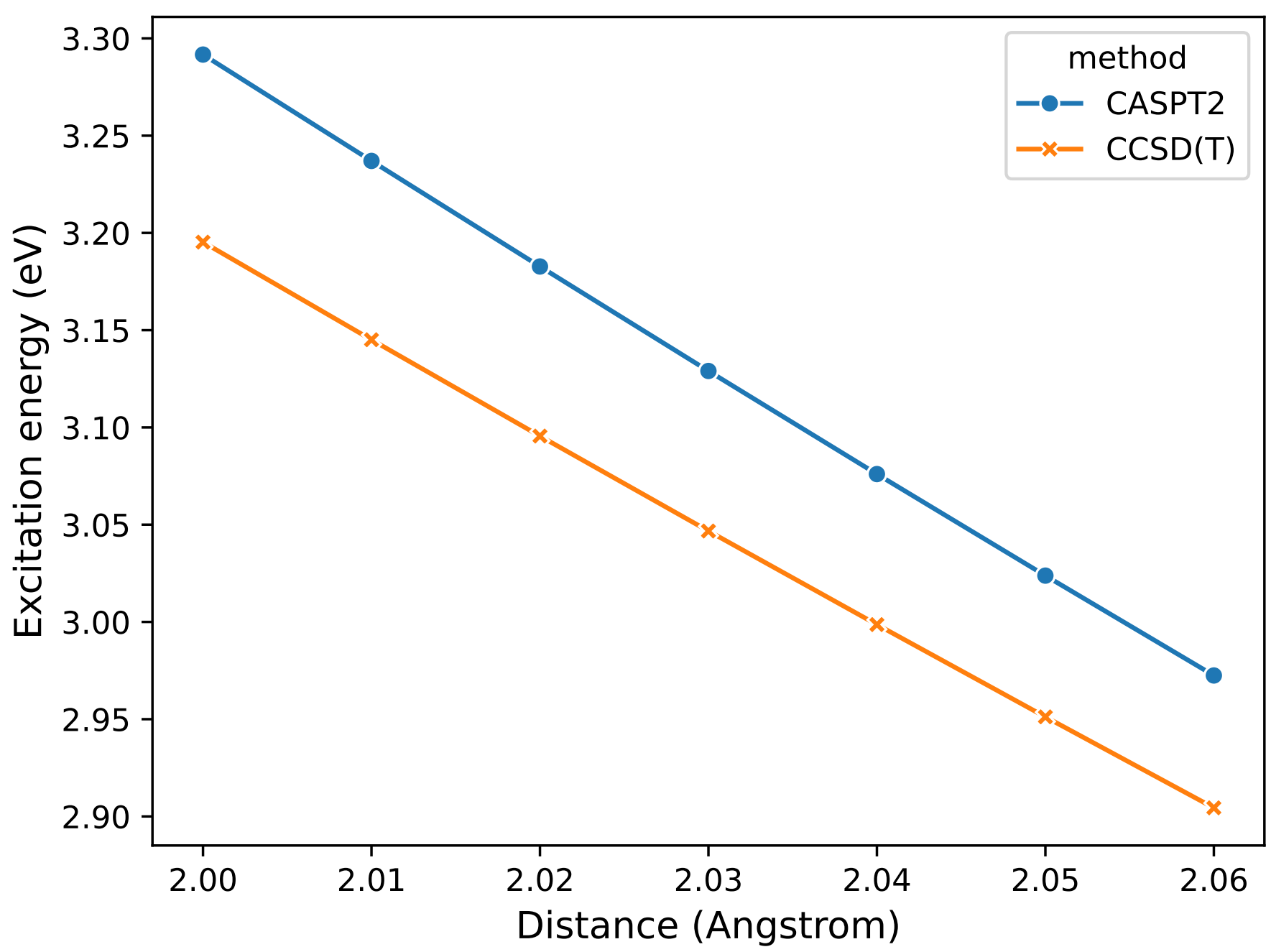

Figure S1: $^2\Pi_g \rightarrow {}^2\Pi_u$ vertical excitation energy of $S_2^{\bullet -}$ as a function of the S–S distance.

Table S12: Calculated S–S bond lengths (Å)

model	theory level	distance
$(S_2^{\bullet -})Na_7$-*sod*	PBE periodic[a]	2.013
$(S_2^{\bullet -}Na_8Cl$-*sod*	PBE periodic[a]	2.017
isolated $S_2^{\bullet -}$	PBE[b]	2.020
	B3LYP[b]	2.019
	CCSD(T)[c]	2.022

[a]See main article for details. [b]ma-def2-TZVP basis. [c]aug-cc-pV(T+d)Z basis.

S2.6 Intra-$^2\Pi_g$ Splitting, SOC Effects, and Orbital Energy Differences

Table S13: Energy Difference Between $^2\Pi_g^{(1),(2)}$ States or $\pi^*_{x,y}$ Orbitals (eV).

quantity	theory level	$S_2^{\bullet -}$	$(S_2^{\bullet -})Na_7$-*sod*			$(S_2^{\bullet -})Na_8Cl$-*sod*		
			**1**S	**1**M	**1**L	**2**S	**2**M	**2**L
ΔE [a]	CASPT2	0	0.01	0.20	0.23	0.13	0.09	0.08
	CASPT2+SOC [c]	0.04	0.04	0.21	0.24	0.14	0.10	0.09
$\Delta\varepsilon$ [b]	CASSCF [d]	0	0.01	0.22	0.25	0.14	0.11	0.11
	CAM-B3LYP [e]	4.34	4.45	4.70	4.73	4.63	4.60	4.59
	B3LYP [e]	1.62	1.66	1.88	1.91	1.82	1.79	1.78
	BP86 [e]	0.14	0.14	0.33	0.36	0.26	0.23	0.22

[a]Energy difference between $^2\Pi_g^{(2)}$ and $^2\Pi_g^{(1)}$ states. [b]Orbital energy difference between π^*_y and π^*_x. [c]Including the SOC within the AMFI approximation using the RASSI method based on CASPT2 energies and CASSCF wave functions. [d]For pseudocanonical orbitals. [e]For spin-unrestricted β orbitals.

S3 Addtional DFT results

Table S14: Comparison of BP86 g tensor principal values obtained for 2M model with various Gaussian (ORCA) and Slater (ADF) basis sets.

Basis	g_x	g_y	g_z	g_{iso}
**2nd order UKS/ORCA**				
def2-TZVP, ma-def2-TZVP^a	2.002	2.031	2.433	2.155
def2-TZVP	2.002	2.031	2.434	2.156
6-311G**	2.002	2.031	2.662	2.232
SVP	2.002	2.029	2.612	2.214
**1st order UKS/ADF**				
TZP, AUG/ATZ2P^a	1.980	2.007	2.436	2.143
TZP	1.967	2.007	2.624	2.199
DZP	1.967	2.007	2.625	2.200
**1st order ROKS/ADF**				
TZP, AUG/ATZ2P^a	0.518	0.519	3.864	1.634
TZP	0.496	0.498	3.870	1.621
DZP	0.505	0.506	3.869	1.627

^{a}Larger basis set, containing diffusion functions, at S atoms only.

Table S15: Comparison of TDDFT excitation energies (in eV) of the most intense $^2\Pi_g \rightarrow {}^2\Pi_u$ type transition obtained without and with the Tamm-Dancoff approximation (TDA).

	$S_2^{\bullet -}$	$(S_2^{\bullet -})Na_7$-*sod*			$(S_2^{\bullet -})Na_8Cl$-*sod*		
		**1**S	**1**M	**1**L	**2**S	**2**M	**2**L
no TDA	3.01	3.10	3.15	3.16	3.24	3.20	3.17
TDA	3.24	3.35	3.40	3.41	3.50	3.50	3.48

S4 Cluster Models of $S_2^{\bullet-}$ Defects in NaCl and KCl

The monovacancy models of $S_2^{\bullet-}$ defects in NaCl and KCl were constructed and optimized in a similar way as in the earlier studies of Stevens et al.[S1,S2]

In the first step, the 88-atom cluster was partially optimized at the DFT level. This 88-atom cluster model contains the $S_2^{\bullet-}$ replacing the central Cl^- and being oriented along the [110] direction as well as ions in shells 1–6 and 9 using the terminology of ref S1 (see tables I and II therein). The smaller 28-atom cluster contains the central $S_2^{\bullet-}$ and shells 1–3. The two clusters thus have stoichiometry $[M_xCl_{x-2}S_2]^+$ where $x = 14$ or 44, M=Na or K. The initial parameters of the regular NaCl/KCl lattices were taken from the literature[S3] ($a_{\mathrm{NaCl}} = 5.6402$ Å, $a_{\mathrm{KCl}} = 6.2931$ Å). The coordinates of the 88-atom cluster were partially optimized using ADF at the SVWN/TZP level (large core), with the scalar relativistic ZORA Hamiltonian, which is the computational protocol similar to that of Stevens et al. Also, in accord with their optimization procedure, only the coordinates of the inner 20 atoms ($S_2^{\bullet-}$ and shells 1 and 2, i.e., six M^+ and twelve Cl^- ions) were relaxed, whereas the coordinates of the remaining atoms were frozen. The MO occupations were controlled to assure that the lobes of the SOMO of the $S_2^{\bullet-}$ moiety was oriented as found by Stevens et al. to be energetically preferred in each lattice, i.e., either perpendicular (NaCl) or parallel (KCl) to the plane defined by S_2 and the four nearest neigbors M^+ ions (see Figure 1 in ref S2).

Subsequently, a smaller 28-atom cluster was cut from the optimized coordinates of the 88-atom cluster and used in the g tensor calculations at the CASPT2 level. The choice of the 28-atom cluster is based on the considerations of computational efficiency as Stevens et al. demonstrated that the 28- and 88-atom clusters lead to similar g tensor predictions at the DFT level.[S2] In the CASPT2 calculations the Douglas–Kroll Hamiltonian was used and the default IPEA shift as well as an imaginary shift of 0.1 a.u were applied. The basis set was chosen consistently with the aT/D one used for UM models, i.e., aug-cc-pV(T+d)Z-DK for S, cc-pVDZ-DK for Na, whereas for K we used jorge-DZP-DKH.[S4] The frozen core was 1s2s2p for S, 1s2s for Na, and 1s2s2p3s for K.

References

(S1) Stevens, F.; Vrielinck, H.; Callens, F.; Pauwels, E.; Waroquier, M. Density-functional study of S_2^- defects in alkali halides. *Phys. Rev. B* **2002**, *66*, 134103.

(S2) Stevens, F.; Van Speybroeck, V.; Pauwels, E.; Vrielinck, H.; Callens, F.; Waroquier, M. Level of theory study of magnetic resonance parameters of chalcogen XY- (X, Y = O, S and Se) defects in alkali halides. *Phys. Chem. Chem. Phys.* **2005**, *7*, 240–249.

(S3) Sirdeshmukh, D. B.; Sirdeshmukh, L.; Subhadra, K. G. *Alkali Halides. A Handbook of Physical Properties*; Springer, 2001.

(S4) Jorge, F. E.; Canal Neto, A.; Camiletti, G. G.; Machado, S. F. Contracted Gaussian basis sets for Douglas-Kroll-Hess calculations: Estimating scalar relativistic effects of some atomic and molecular properties. *J. Chem. Phys.* **2009**, *130*, 064108.
